# A Response Regulator Interfaces between the Frz Chemosensory System and the MglA/MglB GTPase/GAP Module to Regulate Polarity in *Myxococcus xanthus*

**DOI:** 10.1371/journal.pgen.1002951

**Published:** 2012-09-13

**Authors:** Daniela Keilberg, Kristin Wuichet, Florian Drescher, Lotte Søgaard-Andersen

**Affiliations:** Department of Ecophysiology, Max Planck Institute for Terrestrial Microbiology, Marburg, Germany; University of Geneva Medical School, Switzerland

## Abstract

How cells establish and dynamically change polarity are general questions in cell biology. Cells of the rod-shaped bacterium *Myxococcus xanthus* move on surfaces with defined leading and lagging cell poles. Occasionally, cells undergo reversals, which correspond to an inversion of the leading-lagging pole polarity axis. Reversals are induced by the Frz chemosensory system and depend on relocalization of motility proteins between the poles. The Ras-like GTPase MglA localizes to and defines the leading cell pole in the GTP-bound form. MglB, the cognate MglA GTPase activating protein, localizes to and defines the lagging pole. During reversals, MglA-GTP and MglB switch poles and, therefore, dynamically localized motility proteins switch poles. We identified the RomR response regulator, which localizes in a bipolar asymmetric pattern with a large cluster at the lagging pole, as important for motility and reversals. We show that RomR interacts directly with MglA and MglB *in vitro*. Furthermore, RomR, MglA, and MglB affect the localization of each other in all pair-wise directions, suggesting that RomR stimulates motility by promoting correct localization of MglA and MglB in MglA/RomR and MglB/RomR complexes at opposite poles. Moreover, localization analyses suggest that the two RomR complexes mutually exclude each other from their respective poles. We further show that RomR interfaces with FrzZ, the output response regulator of the Frz chemosensory system, to regulate reversals. Thus, RomR serves at the functional interface to connect a classic bacterial signalling module (Frz) to a classic eukaryotic polarity module (MglA/MglB). This modular design is paralleled by the phylogenetic distribution of the proteins, suggesting an evolutionary scheme in which RomR was incorporated into the MglA/MglB module to regulate cell polarity followed by the addition of the Frz system to dynamically regulate cell polarity.

## Author Summary

Most cells are spatially organized with proteins localizing to specific regions. The ability of cells to polarize facilitates many processes including motility. *Myxococcus xanthus* cells move in the direction of their long axis and occasionally change direction of movement by undergoing reversals. Similarly to eukaryotic cells, the leading pole of *M. xanthus* cells is defined by a Ras-like GTPase and the lagging pole by its partner GAP MglB. We show that MglA and MglB localization depends on the RomR protein. RomR recruits MglA to a pole and MglB GAP activity at the lagging pole results in MglA/RomR localizing asymmetrically to the leading pole. Conversely, RomR together with MglB forms a complex that localizes to the lagging pole, and this asymmetry is set up by MglA/RomR at the leading pole. Thus, MglA/RomR and MglB/RomR localize to opposite poles because they exclude each other from the same pole. RomR also interfaces with the Frz chemosensory system that induces reversals. Thus, RomR links the MglA/MglB/RomR polarity module to the Frz signaling module that triggers the inversion of polarity. Phylogenomics suggests an evolutionary scheme in which the MglA/MglB module incorporated RomR early to impart cell polarity while the Frz module was appropriated later on to direct polarity reversals.

## Introduction

The ability of cells to generate polarized distributions of signaling proteins facilitates many biological processes including cell growth, division, differentiation and motility [1]. The spatial confinement of the activity of signaling proteins lays the foundation for processes that require localized protein activity [2], [3]. For instance, directional migration of neutrophils during chemotaxis depends on the dynamic localization of the activated small GTPases Rac and Cdc42 to the front edge of cells where they stimulate the formation of cellular protrusions *via* actin polymerization while Rho activity is spatially confined to the rear end of cells to drive actomyosin contractility with retraction of cellular protrusions [4]. Similarly, chemotaxing cells of *Dictyostelium discoideum* exhibit actin polymerization based cellular protrusions at the front that are dependent of the localization of a small Ras-family GTPase [5]. In both systems, the subcellular localization of small GTPases is highly dynamic and changes in response to environmental conditions [4], [5]. Similar to eukaryotic cells, bacterial cells are highly polarized with proteins localizing to specific subcellular regions, often the cell poles [6]. Two major unresolved questions regarding cell polarity in general are how proteins achieve their correct subcellular localization and how this localization changes dynamically over time. In eukaryotic cells, members of the Ras-superfamily of small, monomeric GTPases have essential functions in regulating dynamic cell polarity [7]. Recent evidence suggests that the function of small Ras-like GTPases in dynamic cell polarity regulation is conserved from eukaryotes to prokaryotes [8].

Ras-like GTPases are binary nucleotide-dependent molecular switches that cycle between an inactive GDP- and an active GTP-bound form [9]. The GTP-bound form interacts with downstream effectors to induce a specific response. Generally, Ras-like GTPases bind nucleotides with high affinities and have low intrinsic GTPase activities [9]. Therefore, cycling between the two nucleotide-bound states depends on two types of regulators: Guanine-nucleotide exchange factors (GEFs), which function as positive regulators by facilitating GDP release and GTP binding, and GTPase activating proteins (GAPs), which function as negative regulators by stimulating the low intrinsic GTPase activity in that way converting the active GTP-bound form to the inactive GDP-bound form [9], [10].

If placed on a surface, cells of the rod-shaped bacterium *Myxococcus xanthus* move in the direction of their long axis with a defined leading and lagging cell pole [8], [11]. Occasionally, however, cells stop and then resume motility in the opposite direction with the old leading pole becoming the new lagging cell pole and *vice versa* [12]. These events are referred to as reversals and at the cellular level a reversal corresponds to an inversion of the leading and lagging cell poles [8], [11]. Recent evidence suggests that a signal transduction module consisting of the small, monomeric Ras-like GTPase MglA and its cognate GAP MglB is at the heart of the regulatory system that controls motility and the cell polarity axis in *M. xanthus*.

*M. xanthus* has two motility systems [11]. The S-motility system depends on type IV pili (T4P), which localize to the leading pole [13]. T4P are thin filaments that undergo cycles of extension, adhesion and retraction [14], [15]. During a retraction, a force is generated that is sufficiently large to pull a cell forward [16], [17]. The A-motility system depends on protein complexes often referred to as focal adhesion complexes (FACs) that are assembled at the leading pole and distributed along the cell body [18]–[20]. Each FAC is thought to consist of a multi-protein complex that spans the cell envelope [19]–[21]. In a moving cell, FACs remain stationary within respect to the surface on which the cell is moving [18]. The two motility systems function independently of each other; however, their activity is coordinated to generate force in the same direction [22].

During a reversal, the polarity of the two motility systems is inverted synchronously. Several T4P proteins localize in clusters at both cell poles and remain stationary during reversals [23]. In contrast, the PilB ATPase, which catalyzes extensions, primarily localizes to the leading pole, and the PilT ATPase, which energizes retractions, primarily localizes to the lagging cell pole. During reversals, PilB and PilT switch poles thereby laying the foundation for the assembly of T4P at the new leading pole [23]. In the case of the A-motility system, several proteins including AglQ, which is part of the A-motility motor [19], [21], AglZ, GltD/AgmU and GltF, which are part of the FACs, localize to the leading cell pole as well as to FACs between reversals [18], [21], [24]. During reversals, the polar protein clusters relocate to the new leading cell pole and, in parallel, the FACs are thought to change polarity [18], [19], [24]. Therefore, at the molecular level, a reversal involves a switch in the polarity of dynamically and polarly localized motility proteins.

MglA functions as a nucleotide-dependent molecular switch to stimulate motility and reversals at the cellular level [25]–[29]. MglA-GTP is the active and MglA-GDP the inactive form [26]–[28]. MglB is the cognate GAP of MglA [26]–[28]. Between reversals MglA-GTP localizes to the leading cell pole while MglA-GDP is distributed uniformly throughout cells [26], [28]. MglB localizes to the lagging cell pole [26], [28]. MglA-GTP generates the output of the MglA/MglB module and MglA-GTP is thought to stimulate motility at the leading cell pole by setting up the correct polarity of dynamically localized motility proteins and by stimulating T4P function and FACs assembly [26], [28]. MglB localizes to the lagging cell pole and excludes MglA-GTP from this pole by converting MglA-GTP to MglA-GDP and, thus, sets up the MglA-GTP asymmetry. In this way, MglA-GTP together with MglB define the leading/lagging polarity between reversals [26], [28].

The Frz chemosensory system induces cellular reversals but is not required for motility *per se* (Blackhart et al., 1985) The Frz system consists of seven protein [30] including the CheA histidine kinase FrzE and the FrzZ response regulator. Genetic and biochemical analyses have demonstrated that FrzZ is phosphorylated by FrzE and FrzZ serves as the output of the Frz system [31], [32]. The effect of Frz on reversals depends on MglA as well as on MglB [26], [28] and signaling by Frz induces the pole switch of MglA-GTP and MglB, thus, giving rise to an inversion of the leading/lagging polarity [26], [28].

We previously showed that the RomR response regulator, which consists of an N-terminal receiver domain and a C-terminal output domain, is essential for A-motility in *M. xanthus* [25]. Full-length RomR localizes in a bipolar, asymmetric pattern with a large cluster at the lagging pole and a small cluster at the leading cell pole. During reversals the polarity of the RomR clusters switches. The activity of response regulators is regulated by phosphorylation of a conserved Asp residue in the receiver domain [33]. A RomR variant in which this Asp residue in the receiver domain is substituted to Glu (RomR^D53E^), which is expected to partially mimic the phosphorylated state [34], causes a hyper-reversing phenotype while a substitution to the non-phosphorylatable Asn (RomR^D53N^) causes a hypo-reversing phenotype [25]. Because a cellular reversal involves the synchronous switch in polarity of both A- and S-motility proteins [25], these observations raised the question of the function of RomR in S-motility and in regulating the reversal frequency.

Here we re-examined the function of RomR in *M. xanthus* motility. We provide evidence that RomR is important for A- as well as for S-motility. Moreover, we show that RomR interacts directly with MglA and MglB. We show that RomR is a polar targeting determinant of MglA-GTP and that RomR together with MglB sets up the asymmetric polar localization of the MglA-GTP defining the leading cell pole. Similarly, we find that RomR sets up the asymmetric localization of MglB and that MglB and RomR are targeted to the opposite cell pole of MglA-GTP in an MglA dependent manner, thereby, defining the lagging cell pole. Thus, correct localization of MglA and MglB to opposite poles depends on RomR. For reversals, we show that RomR functions between the Frz chemosensory module and the MglA/MglB GTPase/GAP module. These observations in combination with phylogenomic analyses suggest that the MglA/MglB module together with RomR constitute the basic module for establishing cell polarity in gliding motility systems, and that the Frz system was incorporated at a later point to allow the dynamic inversion of the polarity axis during reversals. The paper by Zhang et al. [35] describes results similar to those reported here.

## Results

### The RomR response regulator is important for A- and S-motility in *M. xanthus*

We previously demonstrated that RomR is required for A-motility based on the motility phenotype of a *romR* insertion mutant [25]. To determine the function of RomR in S-motility an in-frame deletion of *romR* (Δ*romR*) was generated in the fully motile strain DK1622, which serves as the wild type (WT) in this work. To assess A- and S-motility in the Δ*romR* mutant, motility was tested on soft (0.5%) agar, which is favorable to S-motility, and hard (1.5%) agar, which is favorable to A-motility [36]. S-motility is manifested by colony expansion with the formation of flares of cells at the edge of a colony and A-motility is manifested by colony expansion with the presence of single cells at the edge of a colony.

**Figure 1:**
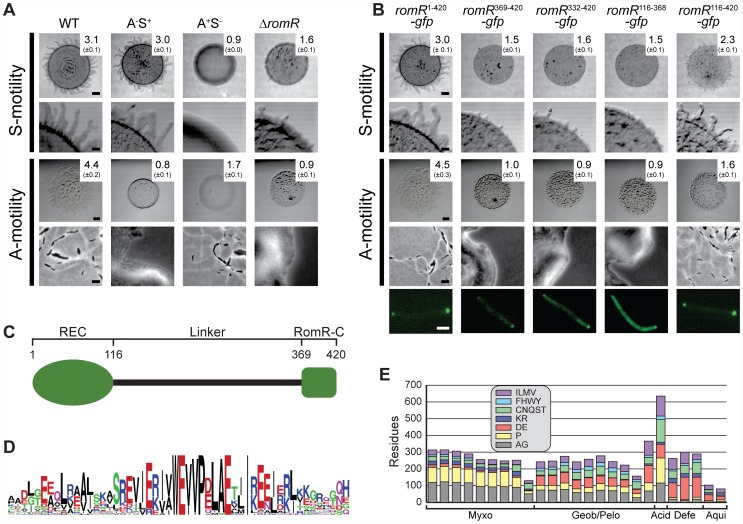
RomR is important for S- as well as for A-motility and contains two pole-targeting determinants. (A) RomR is important for S- as well as for A-motility. The indicated strains were incubated at 32°C for 24 h on 0.5% agar/0.5% CTT medium to score S-motility and 1.5% agar/0.5% CTT medium to score A-motility. S-motility is evaluated by the increase in colony diameter at low magnification (upper row) together with a qualitative analysis of flairs at the colony edge at high magnification (lower row). A-motility is evaluated by the increase in colony diameter at low magnification (upper row) together with a qualitative analysis of single cells at the colony edge at high magnification (lower row). The numbers indicate the increase in colony diameter in mm ± standard deviation after 24 h. Scale bars, 1 mm, 200 µm, 1 mm, and 5 µm from top to bottom row. (B) RomR-C and the linker region are independent pole-targeting determinants and both are required for motility. The top four rows are as described in panel (A). For the experiments in the fifth row, Δ*romR* cells expressing the indicated GFP fusions were transferred from liquid cultures to an agar pad on a slide and imaged by fluorescence microscopy. Scale bar, 2 µm. (C) RomR is composed of three distinct regions: a N-terminal response regulator receiver domain (REC), a conserved C-terminal region unique to RomR (RomR-C), and an unstructured linker region (Linker). Numbers correspond the RomR amino acid sequence from *M. xanthus*. (D) RomR-C is enriched in conserved Glu residues in addition to containing invariant Trp and Pro residues. The sequence logo of RomR-C was built using WebLogo ([67]. (E) The RomR linker displays length and composition in relation to taxonomy. The graph shows the amino acid composition of the linker regions of sequences from Myxococcales (Myxo), Geobacter and Pelobacter species (Geob/Pelo), Acidobacteria (Acid), Deferribacterales (Defe), and Aquificales (Aqui). The amino acids were grouped based on physicochemical properties. Sequences lacking RomR-C were not included in the analysis.

As shown in Figure 1A, the WT DK1622 formed the flares characteristic of S-motility on 0.5% agar, the Δ*romR* mutant was significantly reduced in flare formation and colony expansion, and the A^+^S^−^ control strain DK1300 did not form these flares. On 1.5% agar, the WT displayed the single cell movements characteristic of A-motility at the edge of the colony whereas neither the Δ*romR* mutant nor the A^−^S^+^ control strain DK1217 did. Time-lapse microscopy of Δ*romR* cells at the colony edge on 1.5% agar and on 0.5% agar confirmed that the Δ*romR* cells did not display single cell movements on 1.5% agar and only displayed very limited movements on 0.5% agar (data not shown).

To confirm that the motility defect in the Δ*romR* mutant was caused by lack of RomR, we created a complementation construct in which a functional fusion between full-length RomR and GFP (RomR^1–420^-GFP) was produced from the constitutively active P*pilA* promoter at native levels (Figure S1) [25]. All motility defects were corrected by expression of RomR^1–420^-GFP (Figure 1B) [25]. From these analyses we conclude that RomR is important for S-motility in addition to A-motility.

### Computational and functional analysis of RomR reveals two independent pole-targeting determinants

**Figure 2:**
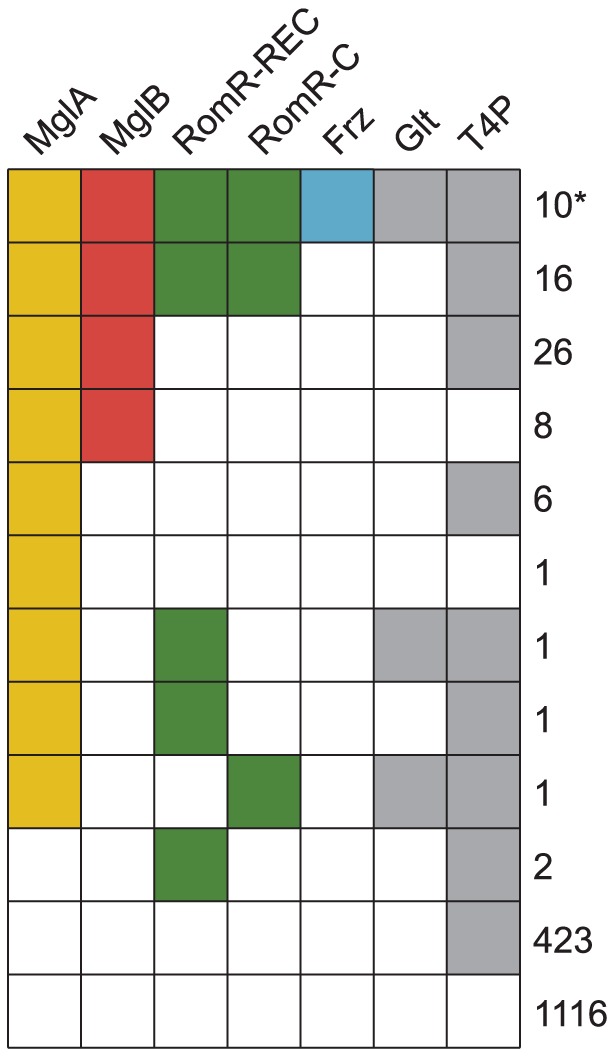
The genomic distributions of RomR and Frz overlap with those of MglA and MglB. Each column represents the presence of absence of MglA, MglB, RomR-REC, RomR-C, Frz, the gliding motility machinery (Glt), or T4P as a colored or white box, respectively. Numbers on the right indicate the number of genomes with a given pattern of co-occurrence. The * indicates the *M. fulvus* genome that contains an incomplete RomR, a complete MglA/MglB system, and Frz system. Analysis of the DNA sequence neighboring its *romR* suggests that the truncation of *romR* is a recent occurrence or the result of a sequencing error because we were able to find neighboring DNA that is nearly identical to the RomR-C encoding portion of *romR* in *M. xanthus*.

Previous characterization of RomR described distinct regions: a response regulator receiver (REC) domain, and an output domain composed of a proline rich (Pro-rich) region and a glutamate (Glu-rich) region [25]. To more universally characterize RomR, we identified its homologs from a set of 1611 prokaryotic genomes. Similarity searches against this genome set using full-length RomR support that it is composed of two conserved regions (Materials and Methods). As expected, one conserved region corresponds to the REC domain. The output domain of RomR comprises two distinct regions: (i) a conserved α-helical C-terminal region (RomR-C) (Figure 1C and 1D) that corresponds to the previously described Glu-rich region and is not homologous to characterized domains; and, (ii) an unstructured region corresponding to the previously described Pro-rich region that links the two conserved regions (Figure 1C). Sequence analysis of all identified homologs showed that most maintain conservation of the RomR-C domain (Figure 1D; Figure 2) while the unstructured linker region was not conserved (Figure 1E). The linker regions show length and composition conservation within taxonomic groups suggesting that they may be associated with lineage-specific functions.

Previous studies [25] have shown that the REC domain alone cannot localize RomR to the poles but is important for reversals. In contrast, the output domain comprising the linker and RomR-C localize polarly and is important for stimulating motility. Informed by the RomR sequence conservation analyses, we carried out a detailed functional analysis of the individual parts of the RomR output domain fused to GFP. As mentioned, full-length RomR fused to GFP (RomR^1–420^-GFP) corrected the motility defects of the Δ*romR* mutant and displayed an asymmetric bipolar localization pattern (Figure 1B) consistent with previous observations [25]. The entire RomR output domain fused to GFP (RomR^116–420^-GFP), RomR-C alone (RomR^369–420^-GFP) and the linker alone (RomR^116–368^-GFP) also localized in an asymmetric bipolar pattern (Figure 1B). However, only the RomR^116–420^-GFP construct partially restored A- and S-motility in the Δ*romR* mutant (Figure 1B). Because the RomR-C construct RomR^369–420^-GFP accumulated at a lower level than native RomR (Figure S1), we examined a RomR-C construct that included a portion of the linker region (RomR^332–420^-GFP). RomR^332–420^-GFP accumulated at a level similar to native RomR (Figure S1) and showed asymmetric bipolar localization (Figure 1B). However, this construct was also unable to complement the motility defects of the Δ*romR* mutant (Figure 1B). From these analyses we conclude that RomR possesses two pole-targeting determinants, the linker region and RomR-C, which are individually sufficient for polar targeting. Moreover, both regions are required for motility.

### RomR co-occurs with the MglA/MglB system

In order to understand the potential interplay between RomR and other systems involved in motility, we compared its phyletic distribution to the distribution of *mglA* and *mglB*, in addition to genes that mark the presence of the Frz system (*frzE*), T4P (*pilT*) and gliding motility (*gltF*) in our genome set. The proteins of interest were identified using BLASTP searches, gene neighborhood analysis, and characteristic features (Materials and Methods). Informed by the analyses on which regions of RomR are conserved and functionally important, we used the REC and RomR-C portions of RomR to identify homologs. RomR was identified in 31 genomes whereas MglA (70 genomes) and MglB (60 genomes) are more widespread (Figure 2). Of the 60 genomes encoding both MglA *and* MglB, 26 also encode a RomR homolog (Figure 2). Thus, with the exception of five genomes, all genomes encoding a RomR homolog also encode MglA and MglB homologs. These five genomes support a close correlation between MglA, MglB and RomR: RomR in these five genomes have lost either REC or RomR-C, and none contain a complete, if any, MglA/MglB system (Figure 2). 10 of the 26 genomes encoding intact RomR proteins also encode a Frz system and all Frz encoding genomes encode homologs of MglA, MglB and RomR. The co-occurrence of Frz with RomR and RomR with MglA and MglB support a functional association between these proteins.

Genes for T4P and gliding motility were found in 476 and 12 genomes, respectively (Figure 2). Generally, MglA, MglB, RomR and Frz encoding genes co-occurred with genes for gliding motility suggesting a functional connection between these proteins. Similarly, all 26 genomes encoding intact genes for MglA, MglB and RomR also contained T4P encoding genes also supporting a functional connection between these genes.

### RomR acts upstream of MglA and MglB in motility and reversals

**Figure 3:**
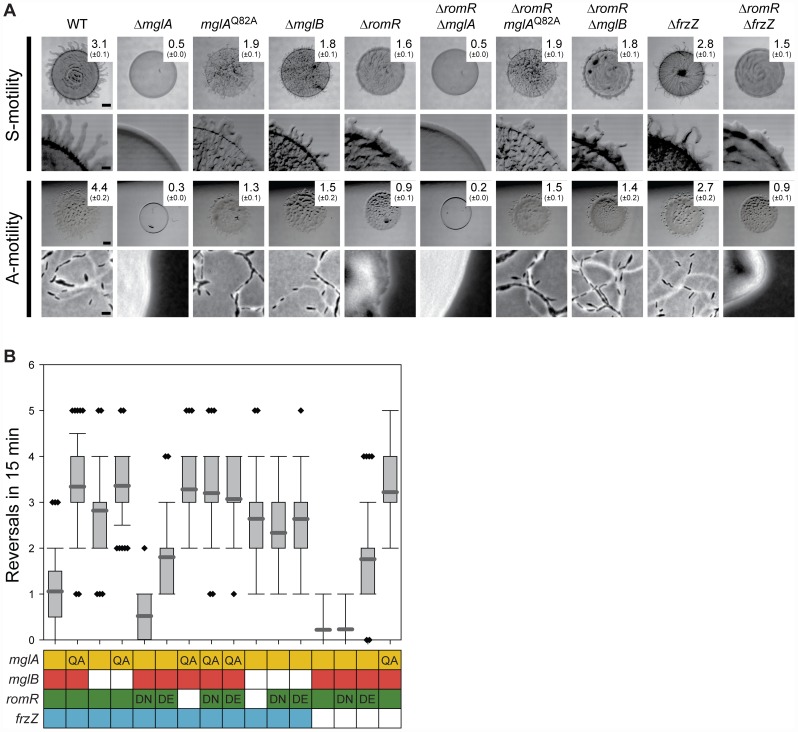
RomR acts upstream of MglA and MglB and downstream of FrzZ. (A) Motility phenotypes of strains of the indicated genotypes. The WT and Δ*romR* images from Figure 1 are included for comparison. Note that hyper- or hypo-reversing mutants expand less than WT colonies due to the abnormal reversal frequency and not due to defects in A- and S-motility [26], [28]. The indicated strains were incubated at 32°C for 24 h on 0.5% agar/0.5% CTT medium and 1.5% agar/0.5% CTT medium to score S- and A-motility, respectively. Motility is evaluated as described in Figure 1A. Scale bars, 1 mm, 200 µm, 1 mm, and 5 µm from top to bottom row. (B) Box plot of reversal frequencies measured in the strains of the indicated genotypes. The boxes below indicate alleles present: Colored, WT; white, in-frame deletion; QA, DN and DE: MglA^Q82A^, RomR^D53N^ and RomR^D53E^. n>50. Cells were transferred from a liquid culture to a thin agar pad, covered with a coverslip and followed by time-lapse microscopy in which cells were imaged at 30-s intervals for 15 min. For each strain, 50 cells were followed. In the box plot, the Y-axis is number of reversals per 15 min, boxes enclose the 25^th^ and 75^th^ percentile with the dark grey line represents the mean, whiskers represent the 10^th^ and 90^th^ percentile, and diamonds outliers.

To map the position of *romR* in the regulatory circuits controlling motility and reversals, we carried out genetic epistasis experiments, using motility and reversal frequencies as readouts for function. Motility assays confirmed that a Δ*mglA* mutant is non-motile [26], [28], unlike the Δ*mglB* or *mglA* ^Q82A^ mutants, which contain MglA locked in the active GTP-bound form, both of which display A- and S-motility and hyper-reverse [27] (Figure 3A and 3B). Next, we deleted *romR* in these three backgrounds to establish the relative order of the genes. The motility assays showed that *mglA*, *mglA* ^Q82A^, and *mglB* are epistatic to *romR* as evidenced by the similar phenotypes shared between the single mutants and corresponding double mutants (Figure 3A). We analyzed the reversal frequencies of single cells in the Δ*romR*, *mglA* ^Q82A^ and Δ*romR*, Δ*mglB* double mutants and found that they displayed hyper-reversing phenotypes similar to *mglA* ^Q82A^ and Δ*mglB* single mutants (Figure 3B), respectively, which further supports the epistasis relationships observed in the motility assays. These data also demonstrate that the *mglA* ^Q82A^ and *mglB* mutations cause a bypass of the motility defects caused by the Δ*romR mutation*.

Previous work suggested that substitutions of D53 in RomR mimics the active phosphorylated state (RomR^D53E^) or the inactive non-phosphorylated state (RomR^D53N^) [25]. We confirmed that RomR^D53N^ and RomR^D53E^ both stimulate motility and that RomR^D53N^ causes a hypo-reversing and RomR^D53E^ a hyper-reversing phenotype (Figure 3B) [25]. Strains containing *romR* ^D53N^ or *romR* ^D53E^ in *mglA* ^Q82A^ or Δ*mglB* mutant backgrounds showed the hyper-reversing phenotypes similar to those of *mglA* ^Q82A^ or Δ*mglB* single mutants, respectively and no additive phenotype was observed (Figure 3B). Thus, the observed epistasis relationships are independent of the activation state of RomR.

The epistasis experiments combining the various *mglA*, *mglB*, and *romR* alleles suggest that *romR* acts in the same genetic pathway as *mglA* and *mglB* to stimulate motility and reversals. Moreover, the data are consistent with *romR* acting upstream of both *mglA* and *mglB* as a positive regulator and inhibitor, respectively. Because MglB is an inhibitor of MglA, an MglB inhibitor is formally similar to an MglA activator. Therefore, these experiments are consistent with three general models for how the effect of RomR on motility and reversals could be accomplished by (i) stimulating MglA; (ii) inhibiting MglB; or, (iii) a combination of the two.

### RomR acts downstream of FrzZ to regulate motility and reversals

Because *frz* acts upstream of *mglA* and *mglB* for reversals [26], [28], we tested whether *romR* lies between *frz* and *mglA* and *mglB*. The FrzZ protein is the direct output of the Frz system [31], [32]. To test the relationship between *frz* and *romR*, we combined a Δ*frzZ* mutation, which causes a hypo-reversing phenotype [32], with different *romR* alleles.

Combining Δ*romR* with Δ*frzZ* did not restore the motility defects caused by the Δ*romR* mutation (Figure 3A). A strain containing *romR* ^D53N^, which is active for motility but not for reversals, and Δ*frzZ* was motile and hypo-reversed similarly to the strains only containing Δ*frzZ* or *romR* ^D53N^ (Figure 3B). A strain containing *romR* ^D53E^, which is active for motility and causes hyper-reversals, and Δ*frzZ* was motile and hyper-reversed with a frequency similar to that caused by *romR* ^D53E^ alone. In agreement with previous observations [26], [28], combining Δ*frzZ* with *mglA* ^Q82A^ resulted in a strain that hyper-reversed with the same frequency as a strain only containing *mglA* ^Q82A^. Thus, MglA is the most downstream part in the reversal circuit. These epistasis experiments suggest that *romR* and *frzZ* act in the same genetic pathway to stimulate reversals. Moreover, the data are consistent with *frzZ* acting upstream of *romR* and with *frzZ* acting as a positive regulator of *romR* for reversals.

### MglA, MglB, and RomR are mutually dependent for correct localization

**Figure 4:**
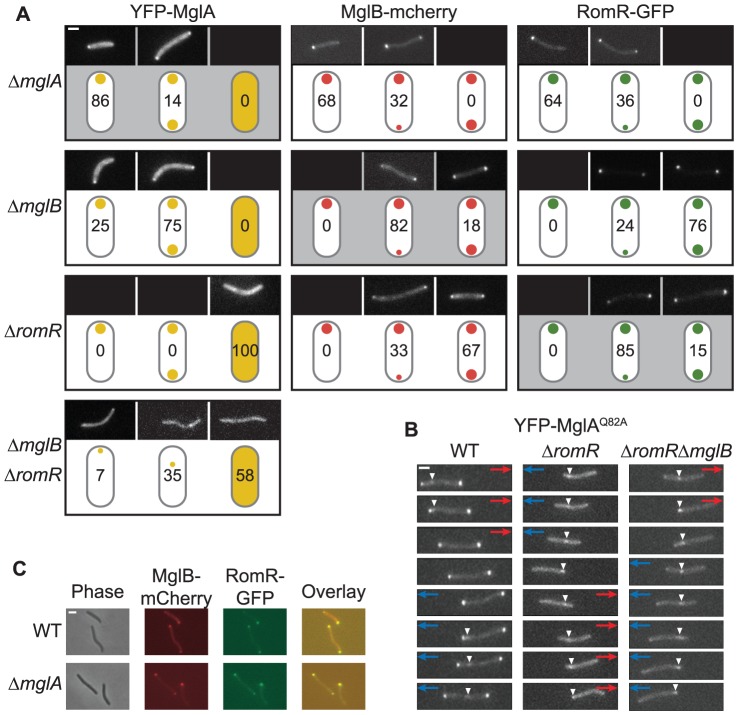
Localization of MglA, MglB, and RomR is mutually dependent. (A) Localization of YFP-MglA, MglB-mCherry and RomR-GFP. Cells were transferred from liquid cultures to a thin agar pad on a microscope slide and imaged by fluorescence microscopy. The localization patterns observed are indicated in the schematics. The ratios between polar signals were calculated to distinguish between unipolar, asymmetric bipolar and symmetric bipolar localization. Schematics highlighted in gray indicate the localization of the fusion proteins in the corresponding in-frame deletion mutants. Representative images of cells are shown for each pattern. Numbers represent % of cells with that pattern. n>200. Scale bar: 2 µm. (B) Time-lapse microscopy of YFP-MglA^Q82A^. Cells of the indicated genotypes and producing YFP-MglA^Q82A^ were treated as in (A) and imaged by time-lapse fluorescence microscopy at 30-s intervals. Red and blue arrows indicate opposite directions of movement. White arrowheads indicate the oscillating cluster formed by YFP-MglA^Q82A^. Scale bar: 2 µm. (C) MglB and RomR colocalize. Cells expressing MglB-mCherry and RomR-GFP were treated as in (A). Right column, overlay of RomR-GFP and MglB-mCherry. Scale bar: 2 µm.

The performed epistasis analyses support that MglA, MglB, RomR and FrzZ are part of a signaling network that regulates motility and reversals in *M. xanthus*. Previous studies of MglA, MglB, and RomR have demonstrated that all three proteins localize polarly. To understand how MglA, MglB and RomR interact to stimulate motility and reversals, we systematically determined the localization of MglA, MglB and RomR in the presence and absence of each other. We have been unable to construct a functional FrzZ fusion protein; therefore, FrzZ was excluded from these analyzes. First, MglA, MglB and RomR were localized using active fluorescent fusion proteins expressed at native levels in strains deleted for the relevant native copies [25], [26] (Figure S2). As previously observed, MglA predominantly localizes in a unipolar pattern, whereas MglB and RomR predominantly localize in a bipolar asymmetric pattern [26]–[28] (Figure 4A).

Next, we analyzed the localization of each protein in the absence of one other. We confirmed that MglA localization changes from unipolar to a predominantly bipolar symmetric pattern in the absence of MglB [26]–[28] (Figure 4A). In contrast, we found that MglA localized diffusely throughout the cytoplasm in the absence of RomR. When examining MglB localization, we found that MglB shifts from a predominantly bipolar asymmetric pattern to a bipolar symmetric pattern in the absence of RomR and a unipolar pattern in the absence of MglA (Figure 4A). RomR localization patterns showed a similar shift from predominantly bipolar asymmetric to unipolar in the absence of MglA, whereas it became more bipolar symmetric in the absence of MglB (Figure 4A). Therefore, all three proteins are mutually dependent for correct localization in all three pair-wise directions.

### RomR is a polar targeting determinant of MglA

Lack of RomR causes diffuse localization of MglA. Because MglA-GDP localizes in a diffuse pattern [26] and MglA-GTP localizes polarly, we thought of four possibilities for how RomR could stimulate polar localization of MglA-GTP: (i) RomR acts as a GEF; (ii) RomR inhibits MglB GAP activity; (iii) RomR is an MglA polar targeting determinant; or, (iv) combinations of these activities. To explore these possibilities, we determined the localization of YFP-MglA^Q82A^, which is locked in the GTP-bound form and localizes in a bipolar pattern and with a central oscillating cluster in a Δ*mglA* mutant [27] (Figure 4B). In the absence of MglB, YFP-MglA^Q82A^ localizes as in the Δ*mglA mglB* ^+^ mutant [27]. In contrast, in the absence of RomR, YFP-MglA^Q82A^ only localized to the central oscillating cluster (Figure 4B). Similarly, in the absence of RomR and MglB, YFP-MglA^Q82A^ only localized to the central oscillating cluster (Figure 4B). Finally, we observed that in the absence of both RomR and MglB, YFP-MglA was primarily diffuse or formed a non-polar cluster and rarely formed polar clusters (Figure 4A). These localization patterns suggest that one function of RomR is as a direct polar targeting determinant of MglA; however, the data does not rule out the possibility that RomR may also regulate the nucleotide-bound state of MglA.

### RomR colocalizes with MglB

MglB-mCherry and RomR-GFP show a similar localization pattern in WT and in the Δ*mglA* mutant (Figure 4A). To determine whether MglB-mCherry and RomR-GFP colocalize, we constructed a strain expressing both fusion proteins. Consistent with the observations that RomR as well as MglB in moving cells localize with the large cluster at the lagging cell pole [26], [28], the two proteins colocalized in *mglA* ^+^ cells with a bipolar, asymmetric localization (Figure 4C). MglB-mCherry and RomR-GFP also colocalized in the absence of MglA (Figure 4C). We previously showed that the unipolar RomR cluster in the Δ*mglA* mutant is at the pole containing T4P [25] and, thus, RomR and MglB both localize at the “wrong” pole in the absence of MglA. This observation in combination with the observation that in the absence of RomR, MglB becomes more symmetric and *vice versa* (Figure 4A) suggest that MglB and RomR depend on each other for bipolar, asymmetric localization and that MglA is important for establishing this pattern.

### RomR interacts directly with MglA as well as with MglB

**Figure 5:**
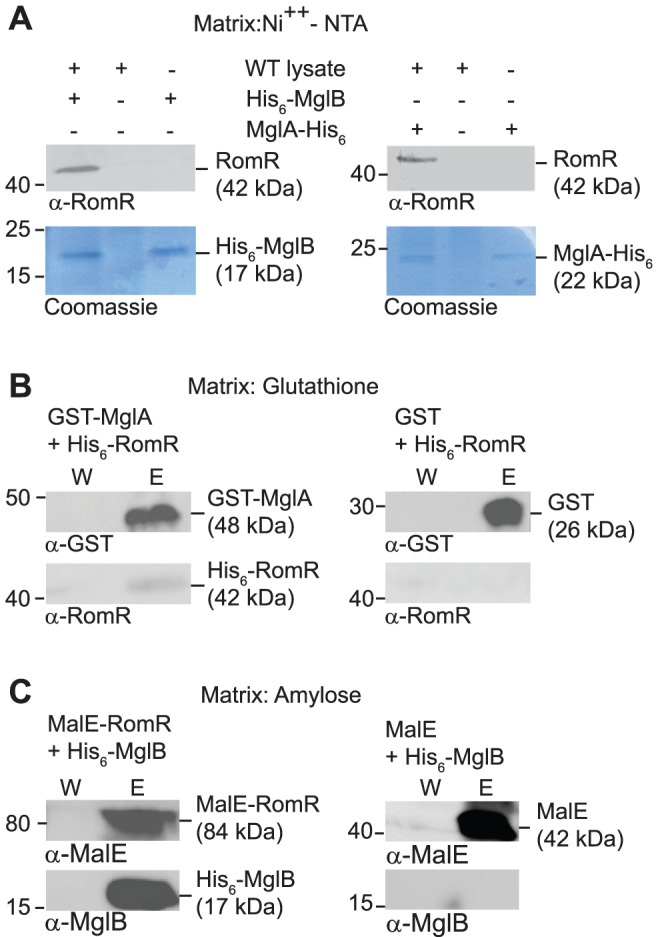
RomR interacts directly with MglA and MglB. (A) RomR interacts with His_6_-MglB and MglA-His_6_. WT *M. xanthus* cell extract was applied to a Ni^++^-NTA-agarose column with or without bound His_6_-MglB (left) and with or without MglA-His_6_ (right). Eluted proteins were separated by SDS-PAGE and visualized in immunoblots with α-RomR (upper panels) or by Coomassie Brilliant Blue R-250 staining (lower panels). Positions of His_6_-MglB, MglA-His_6_ and RomR including their calculated molecular masses are indicated. Migration of molecular weight markers in kDa is indicated on the left. (B) RomR interacts directly with MglA. Purified His_6_-RomR was applied to a glutathione-agarose column with bound GST-MglA (left) or with bound GST (right). Shown are proteins from the last wash fraction before elution (W) and from the elution (E). Eluted proteins were separated by SDS-PAGE and visualized in immunoblots with α-GST (upper panels) and α-RomR (lower panels). GST- MglA and His_6_-RomR including their calculated molecular masses are indicated. Migration of molecular weight markers in kDa is indicated on the left. (C) RomR interacts directly with MglB. Purified His_6_-MglB was applied to an amylose-agarose column with bound MalE-RomR (left) or with bound MalE (right). Shown are proteins from the last wash fraction before elution (W) and from the elution (E). Eluted proteins were separated by SDS-PAGE and visualized in immunoblots with α-MalE (upper panels) and α-MglB (lower panels). MalE-RomR, His_6_-MglB and MalE including their calculated molecular masses are indicated. Migration of molecular weight markers in kDa is indicated on the left.

To test whether RomR interacts directly with MglA and/or MglB, we performed pull-down experiments. To this end we purified N-terminal His_6_-tagged MglB (His_6_-MglB) and C-terminal His_6_-tagged MglA (MglA-His_6_). When bound to a Ni^2+^-NTA-agarose matrix His_6_-MglB interacted with RomR in total cell extracts of WT *M. xanthus* as determined using α-RomR antibodies (Figure 5A). Similarly, when MglA-His_6_ was bound to the Ni^2+^-NTA-agarose matrix, it interacted with RomR in total cell extracts of WT *M. xanthus* (Figure 5A).

To discriminate between direct and indirect interactions between the three proteins, we purified N-terminally His_6_-tagged RomR (His_6_-RomR) and MalE-tagged RomR (MalE-RomR) and N-terminally GST-tagged MglA (GST-MglA). As shown in Figure 5B, GST-MglA bound to a glutathione-agarose column interacted with His_6_-RomR. In control experiments with purified GST, His_6_-RomR was not pulled-down. In a separate control experiment, a His_6_-PilP protein was not pulled-down by GST-MglA (data not shown). Thus, the interaction between GST-MglA and His_6_-RomR is specific and direct.

In a separate set of experiments, MalE-RomR bound to an amylose matrix interacted with His_6_-MglB (Figure 5C) but not with a His_6_-PilP control protein (data not shown). Moreover, purified MalE protein did not interact with His_6_-MglB. Thus, MalE-RomR interacts specifically and directly with MglB.

## Discussion

### Motility is regulated by two distinct signaling modules

Here we report that *M. xanthus* motility is stimulated and regulated by two modules of signaling proteins: a polarity module consisting of the response regulator RomR, the small GTPase MglA, and the MglA GAP MglB, and a polarity inversion module consisting of the Frz chemosensory system with its output response regulator FrzZ. While the RomR/MglA/MglB polarity module is important for motility, the Frz polarity inversion module interfaces with the RomR/MglA/MglB module at the level of RomR to regulate motility by regulating the reversal frequency. Here we focused on understanding the network topology of the polarity module and how it interfaces with the polarity inversion module to ultimately regulate motility.

**Figure 6:**
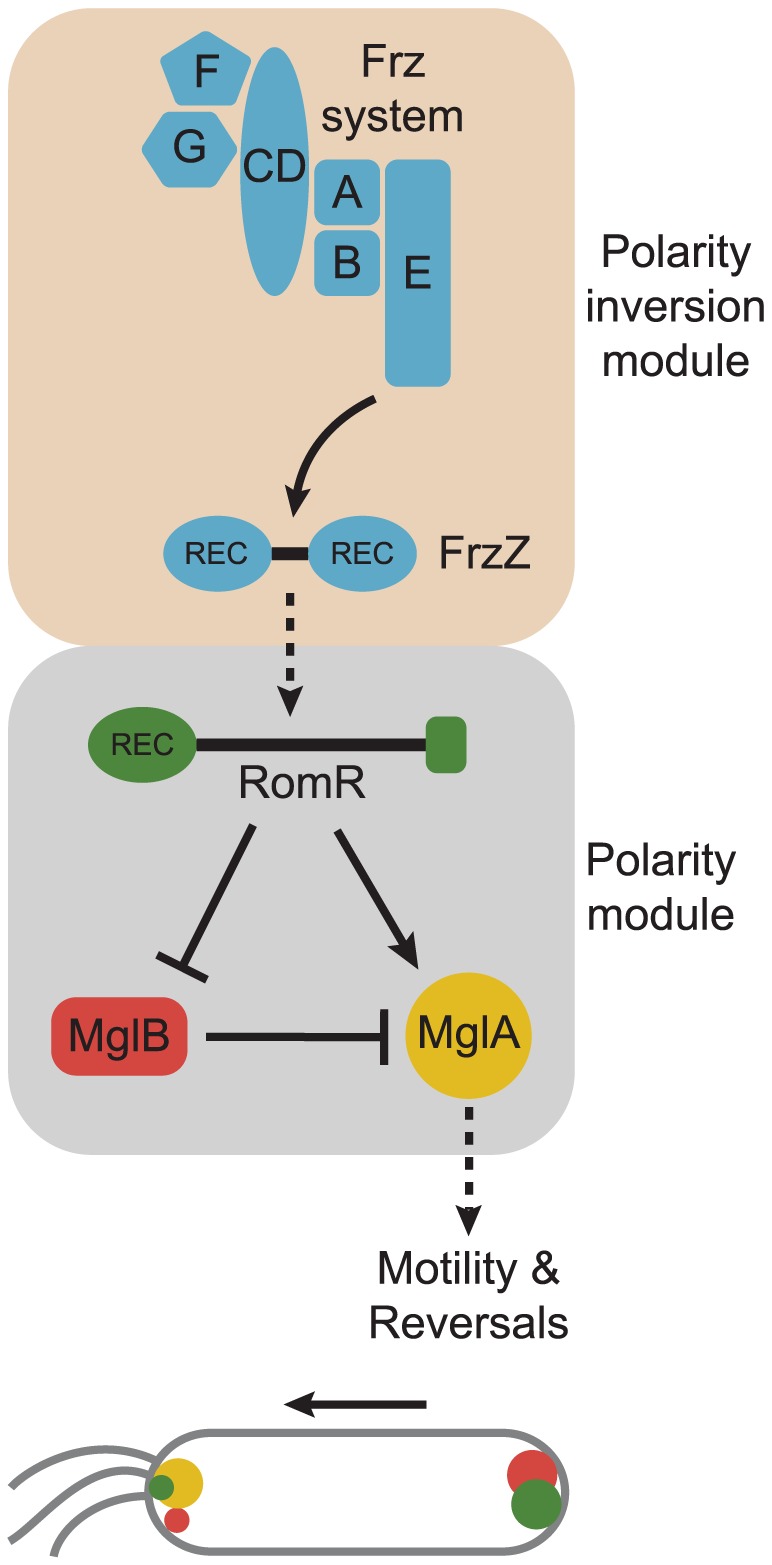
Model for dynamic cell polarity regulation in *M. xanthus*. The upper schematic illustrates the interactions between the Frz chemosensory module for polarity inversion (light brown background) and the MglA/MglB/RomR polarity module (light grey background). Arrows and T-bars indicate direct interactions and the stippled arrow that the molecular mechanism underlying this interaction is not known. The lower schematic illustrates the localization of the MglA, MglB and RomR proteins in a cell moving in the direction indicated by the arrow and with T4P at the leading pole. The color code is as in the upper panel.

MglA-GTP functions to stimulate motility and reversals in the absence of MglB whereas the opposite is not the case. Therefore, MglA-GTP is the output of the MglA/MglB GTPase/GAP module (Figure 6). RomR, MglA-GTP and MglB are all polarly localized whereas MglA-GDP is not. We found that correct localization of the three proteins is mutually dependent in all three pair-wise interactions. Moreover, pull-down experiments using purified proteins and WT *M. xanthus* cell extracts or direct interactions studies with purified proteins together with previous results [26]–[28] show that the three proteins interact in all three pair-wise directions.

Based on the findings from the interaction and localization analyses, we suggest that RomR targets MglA-GTP to both poles and that MglB at the lagging cell pole is important for establishing the MglA-GTP/RomR asymmetry by means of its GAP activity. Thus, RomR is part of a MglA-GTP/RomR complex at the leading cell pole. Interestingly, MglA is neither polarly localized in the Δ*romR* mutant nor in the Δ*romR*, Δ*mglB* double mutant; however, the Δ*romR* mutant is strongly reduced in motility whereas the Δ*romR*, Δ*mglB* mutant is motile. We suggest that the crucial difference between the two strains is the presence and absence of the MglB GAP activity. In the Δ*romR* mutant, MglB is bipolar symmetrical and, consequently, the GAP activity is not confined spatially to a single pole and, therefore, MglA-GTP would be low. On the other hand, the Δ*romR*, Δ*mglB* mutant would not have GAP activity and, therefore, a sufficient level of MglA-GTP may accumulate to stimulate motility. In the Δ*romR* Δ*mglB* mutant, MglA is not polarly localized; nevertheless, this mutant is motile. Therefore, polar localization of MglA is not a strict requirement for motility.

The localization and interaction data suggest that MglB and RomR form a complex that is essential for establishing the bipolar asymmetric localization of the two proteins and that this asymmetry is established in an MglA-GTP/RomR-dependent manner. In total, these interactions generate a mutually-dependent circuit for asymmetric localization of the three proteins: (i) RomR targets MglA-GTP to the poles in the MglA-GTP/RomR complex, (ii) the MglB/RomR complex is essential for establishing the MglA-GTP/RomR asymmetry by means of the MglB GAP activity, and (iii) MglA-GTP/RomR is essential for establishing the MglB/RomR asymmetry.

Combining the localization and interaction data with the results of the epistasis experiments using motility and reversals as readouts, we suggest that between reversals RomR functions as a positive regulator of MglA by targeting MglA-GTP to the poles in the MglA-GTP/RomR complex and that RomR inhibits MglB (and in that way also activates MglA) by formation of the MglB/RomR complex that is targeted to the lagging cell pole in an MglA-GTP/RomR-dependent manner (Figure 6). The identification of the MglA/MglB/RomR polarity module for stimulation of motility provides a conceptual framework for detailed biochemical experiments to address whether RomR acts as a GEF on MglA and/or regulates MglB GAP activity.

The output of the Frz polarity inversion module is the FrzZ response regulator and the reversal-inducing activity of the Frz system depends on phosphorylation of FrzZ [31], [32]. Similarly, our data suggest that reversals are induced by RomR phosphorylation. Interestingly, the reversal frequency of the *romR* ^D53E^ mutant is two-fold lower than in the Δ*mglB* and *mglA* ^Q82A^ mutants possibly reflecting that RomR^D53E^ is not a perfect mimic of phosphorylated RomR. Alternatively, the FrzZ signal is channeled to MglA and MglB in a pathway that is independent of RomR. Given that the *romR* ^D53N^ mutant has the same low reversal frequency as the Δ*frzZ* mutant, we favor the former model. By combining our genetic data with previously published data [31], [32], we suggest that phosphorylated FrzZ acts as a positive regulator of RomR and that this effect likely depends on phosphorylation of RomR. In this model, RomR acts at the interface between the Frz polarity inversion module and the MglA/MglB/RomR polarity module (Figure 6).

This potential phosphorylation of RomR by an unknown mechanism induces a switch in the polarity of the MglA, MglB and RomR proteins. RomR^D53N^ and RomR^D53E^ both localize in a bipolar asymmetric pattern [25] suggesting that the effect of RomR phosphorylation is not directly on its polar localization or release. Clearly, detailed biochemical experiments will be needed to elucidate the interaction between FrzZ/RomR, MglA/RomR and MglB/RomR and how these interactions depend on the phosphorylation status of RomR. Our preliminary results suggest that the FrzE kinase does not phosphorylate RomR *in vitro* (Keilberg, D. unpubl). The widespread distribution of MglA, MglB and RomR in organisms lacking the Frz system suggests that the RomR phosphorylation state could be regulated by other mechanisms. Phosphorylated FrzZ could activate a yet to be identified histidine protein kinase, which would subsequently be involved in RomR phosphorylation, as has been described for the single receiver domain response regulator DivK in the activation of the histidine protein kinases DivJ and PleC in *Caulobacter crescentus* [37]. Alternatively, FrzZ and RomR may be part of a phosphorelay in which the phosphoryl-group would be transferred from FrzZ to RomR via a histidine-phosphotransfer protein as has been described for other phosphorelays [38]. Future experiments will be directed at distinguishing between these possibilities.

### Polarity and modularity as themes in signal transduction

In bacteria many proteins localize to the cell poles [6]. Sophisticated mechanisms are employed to bacteria to facilitate polar binding of proteins: This polar localization can be mediated by *trans*-acting polar targeting factors as in the case of PopZ, which interacts directly with ParB and targets it to the cell poles in *C. crescentus* [39], [40]. Alternatively, proteins may localize to the cell poles based on recognition of membrane curvature as proposed for some peripheral membrane proteins in *Bacillus subtilis* [41], [42]. Understanding how MglA, MglB, and RomR recognize the cell poles will add to our understanding of the diversity of protein localization mechanisms and potential common traits they share.

The modular design of the regulatory circuits involved in motility and its control in *M. xanthus* are paralleled by the phylogenetic distribution of MglA, MglB, RomR and of the Frz system. With the exception of the *M. xanthus* proteins, the functions of these proteins are not known. Based on the analyses of the *M. xanthus* proteins, we suggest that MglA and MglB together with RomR may constitute a module for the spatial deployment of proteins, i.e. regulation of cell polarity (and giving rise to unidirectional cell movements without reversals in *M. xanthus*). Subsequently, the Frz chemosensory module was incorporated by some of these systems to establish a scheme for the dynamic temporal control of cell polarity (and giving rise to the irregular reversals observed in extant *M. xanthus*). As outlined in [43]–[46] the high degree of modularity of signaling systems makes these systems more evolvable in part because combining and integrating different modules allow for the comparatively simple evolution of signaling units with novel properties compared to building such units from scratch. The evolutionary scenario outlined here is in agreement with these concepts.

## Materials and Methods

### Cell growth and construction of strains

Plasmids were propagated in *E. coli* TOP10 (F^−^, *mcrA*, Δ(*mrr-hsd*RMS-*mcr*BC), φ80*lac*ZΔM15, Δ*lac*X74, *deo*R, *rec*A1, *ara*D139, Δ(*ara-leu*)7679, *gal*U, *gal*K, *rps*L, *end*A1, *nup*G) unless otherwise stated. *E. coli* cells were grown in LB or on plates containing LB supplemented with 1.5% agar at 37°C with added antibiotics if appropriate [46]. DK1622 was used as WT *M. xanthus* strain throughout and all *M. xanthus* strains used are derivatives of DK1622. *M. xanthus* strains used are listed in Table 1. Plasmids are listed in Table S1. Plasmid constructions are described in Text S1. Primers used are listed in Table S2. All DNA fragments generated by PCR were verified by sequencing. All *M. xanthus* strains constructed were confirmed by PCR. Plasmids were integrated by site specific recombination at the Mx8 *attB* site or by homologous recombination at the native site. The in-frame deletions of *frzZ* (Δ*frzZ*) and *romR* (Δ*romR*) were generated as described [47] using pFD1 and pSL37, respectively. *M. xanthus* strains were grown at 32°C in 1% CTT broth [48] or on CTT agar plates supplemented with 1.5% agar. Kanamycin (50 µg/ml) or oxytetracycline (10 µg/ml) was added when appropriate.

**Table 1:** *M. xanthus* strains used in this study.

Strain	Genotype	Source
DK1622	Wild type	[68]
DK1217	*aglB1*	[69]
DK1300	*sglG1*	[69]
SA3387	Δ*mglB*	[26]
SA4420	Δ*mglA*	[26]
SA3833	*mglA* ^Q82A^	This work
SA3995	*mglA* ^Q82A^, Δ*romR*	This work
SA4440	Δ*mglA/PpilA-yfp-mglA* (pSL60)	[26]
SA3831	Δ*mglB*, Δ*mglA/PpilA-yfp-mglA* ^Q82A^ (pTS10)	[26]
SA3385	Δ*mglB*, Δ*mglA/PpilA-yfp-mglA* (pSL60)	[26]
SA3300	Δ*romR*	This work
SA3916	Δ*romR/PpilA-romR-GFP* (pGFy177)	This work
SA3980	Δ*romR/PpilA-romR* ^D53N^ *-GFP* (pGFy178)	This work
SA3981	Δ*romR/PpilA-romR* ^D53E^ *-GFP* (pGFy166)	This work
SA3906	Δ*romR/PpilA-romR* ^116–420^-*GFP* (pSH1202)	This work
SA3903	Δ*romR/PpilA-romR* ^369–420^-*GFP* (pDK3)	This work
SA3904	Δ*romR/PpilA-romR* ^116–368^-*GFP* (pDK4)	This work
SA3905	Δ*romR/PpilA-romR* ^332–420^ *-GFP* (pDK5)	This work
SA3906	Δ*romR/PpilA-romR* ^116–420^ *-GFP* (pDK6)	This work
SA3937	Δ*romR/PpilA-yfp-mglA* ^Q82A^ (pTS10)	This work
SA3982	*mglA* ^Q82A^, Δ*romR/PpilA-romR* ^D53N^-*GFP* (pGFy178)	This work
SA3983	*mglA* ^Q82A^, Δ*romR/PpilA-romR* ^D53E^-*GFP* (pGFy166)	This work
SA3936	Δ*mglB*, Δ*romR*	This work
SA3984	Δ*mglA*, Δ*romR*	This work
SA3985	Δ*frzZ*	This work
SA3986	Δ*frzZ*, Δ*romR*	This work
SA3987	Δ*frzZ*, Δ*romR/PpilA-romR* ^D53N^ *-GFP* (pGFy178)	This work
SA3988	Δ*frzZ*, Δ*romR/PpilA-romR* ^D53E^ *-GFP* (pGFy166)	This work
SA3989	Δ*mglB*, Δ*romR/PpilA-romR* ^D53N^ *-GFP* (pGFy178)	This work
SA3990	Δ*mglB*, Δ*romR/PpilA-romR* ^D53E^ *-GFP* (pGFy166)	This work
SA3991	Δ*frzZ/PpilA-YFP-mglA* ^Q82A^ (pTS10)	This work
SA3963	*mglB-mCherry*	This work
SA3971	Δ*mglA, mglB-mCherry*	This work
SA3966	Δ*romR, mglB-mCherry*	This work
SA3992	Δ*mglB*, Δ*romR/PpilA-romR-GFP* (pGFy177)	This work
SA3993	Δ*mglA*, Δ*romR/PpilA-YFP-mglA* (pSL60)	This work
SA3994	Δ*mglA*, Δ*romR/PpilA-romR-GFP* (pGFy177)	This work
SA3978	Δ*romR*, *mglB-mcherry/PpilA-romR-GFP* (pGFy177)	This work
SA3979	Δ*romR*, Δ*mglA*, *mglB-mcherry/PpilA-romR-GFP* (pGFy177)	This work
SA3829	Δ*mglA/PpilA-yfp-mglA* ^Q82A^ (pTS10)	[26]
SA3996	Δ*romR*, *ΔmglA/PpilA-yfp-mglA* ^Q82A^ (pTS10)	This work
SA3997	Δ*romR*, Δ*mglB*, *ΔmglA/PpilA-yfp-mglA* ^Q82A^ (pTS10)	This work
SA3998	Δ*romR*, Δ*mglB*, Δ*mglA/PpilA-YFP-mglA* (pSL60)	This work

1 Plasmids in brackets were integrated at the Mx8 *attB* site and express the listed fusion protein from the *pilA* promoter (P*pilA*).

### Motility assays

Cells were grown to a cell density of 7×10^8^ cells/ml, harvested and resuspended in 1% CTT to a calculated density of 7 ×10^9^ cells/ml. 5 µl aliquots of cells were placed on 0.5% and 1.5% agar supplemented with 0.5% CTT and incubated at 32°C. After 24 h, colony edges were observed using a Leica MZ8 stereomicroscope or a Leica IMB/E inverted microscope and visualized using Leica DFC280 and DFC350FX CCD cameras, respectively. To quantify differences in motility, the increase in colony diameter after 24 h was determined. Briefly, the diameter of each colony was measured at two positions at 0 and 24 h. The increase in colony diameter was calculated by subtraction of the size at 0 h from the size at 24 h. Colony diameters were measured for three colonies per strain.

### Microscopy and determination of reversal frequency

For microscopy, *M. xanthus* cells were placed on a thin 1% agar-pad buffered with A50 buffer (10 mM MOPS pH 7.2, 10 mM CaCl_2_, 10 mM MgCl_2_, 50 mM NaCl) on a glass slide and immediately covered with a coverslip, and then imaged. Quantification of fluorescence signals was done as follows. The integrated fluorescence intensity of polar clusters and of a similar cytoplasmic region was measured using the region measurement tool in Metamorph 7.7. The intensity of the cytoplasmic region was subtracted from the intensity of the polar cluster. These corrected intensities of the polar clusters were used to calculate the ratios between the polar signals in individual cells. If the ratio is ≤2.0, the localization is defined as bipolar symmetric, if the ratio is ≥2.1 and ≤10.0 the localization is defined as bipolar asymmetric, and if the ratio was ≥10.1 the localization is defined as unipolar. For each strain 200 cells were analyzed. For time-lapse microscopy, cells were recorded at 30-s intervals for 15 min. Images were recorded and processed with Leica FW4000 V1.2.1 or Image Pro 6.2 (MediaCybernetics) software. Processed images were visualized using Metamorph (Molecular Devices). Reversals were counted for >50 cells of each strain followed for 15 minutes and displayed in a Box plot.

### Pull-down experiments

Proteins were purified as described in Text S1. 0.5 mg of purified His_6_-MglB or MglA-His_6_ in buffer H (50 mM NaH_2_PO_4_ pH 8.0, 300 mM NaCl, 10 mM imidazole) was applied to a Ni^2+^-NTA-agarose column (Macherey-Nagel). *M. xanthus* cell lysate was prepared as follows: 200 ml of exponentially growing WT cells at a cell density of 7×10^8^ cells/ml were harvested, resuspended in buffer H in the presence of proteases inhibitors (Roche) and lysed by sonication. Cell debris was removed by centrifugation at 4700×g for 20 min, 4°C and the cell-free supernatant applied to the Ni^2+^-NTA-agarose column with or without bound His_6_-MglB or MglA-His_6_. After two washing steps with each 10 column volumes of the buffer H, bound proteins were eluted with buffer H supplemented with 250 mM imidazole. Proteins eluted from the columns were analyzed by two methods: SDS-PAGE and gels stained with Coomassie Brilliant Blue R-250 and SDS-PAGE with immunoblot analysis using α-RomR antibodies [25].

To test for direct protein-protein interactions, 0.2 mg of purified prey protein (His_6_-RomR or His_6_-MglB or as a negative control His_6_-PilP) was mixed with 0.2 mg of purified bait protein (GST-MglA or MalE-RomR) and as a control with 0.2 mg of GST or MalE, respectively. Proteins were incubated with 0.5 ml sepharose beads (for MalE-tagged proteins: amylose beads; for GST-tagged proteins: glutathione beads) in buffer D (50 mM NaH_2_PO_4_ pH 8.0, 300 mM NaCl) for 5 h, 4°C. After washing the beads with 25 column volumes of buffer D, the elutions were performed with buffer D supplemented with 10 mM glutathione for GST-tagged proteins, and with 10 mM maltose for MalE-tagged proteins. Proteins eluted from the columns were analyzed by immunoblot analysis using α-GST antibodies (Biolabs), α-MalE antibodies (Biolabs), α-RomR antibodies [25] and α-MglB antibodies [26]. Immunoblots were carried out as described [46].

### Software versions and default settings

The following software packages were used with the described settings unless otherwise specified. The HMMER3 software package [49] was used in conjunction with the Pfam26 domain library [50] for domain architecture analysis with default gathering thresholds. In the event of domain overlaps, the highest scoring domain model was chosen for the final architecture. The JackHMMER method [51] was used for iterative similarity searches with a 0.0001 e-value inclusion threshold. For non-iterative similarity searches, we used BLASTP from the BLAST+ software package version 2.2.26 [52] and considered hits with e-values of 0.0001 or lower to be significant unless otherwise specified. Multiple sequence alignments were built using the l-ins-i algorithm of the MAFFT version 6.864b software package [53]. Phylogenetic trees were constructed using FastTree version 2.1.4 [54] with default settings or PhyML version 3.0 [55] with empirical frequencies and SPR topology searches. Secondary structure was predicted using the Jpred3 webserver [56].

### Genome set

All complete prokaryotic genomes 1609 were downloaded from the NCBI Refseq [57] database on April 4^th^, 2012. Due to our specific interest in Myxococcales, we also included the complete genomes of *Stigmatella aurantiaca* [58] and *Corallococcus coralloides* [59] from GenBank [60] as they were not yet available in Refseq at the time of genome collection.

### Identification of MglA and MglB sequences

The MglA and MglB sequences from *M. xanthus* (MXAN_1925 and MXAN_1926, respectively) were used in BLASTP queries against the genome set. All significant sequence hits were aligned using MAFFT and the core regions were extracted and used to build phylogenetic trees with FastTree. The tree representing 134 putative MglA homologs showed a distinct subfamily of 113 sequences that is associated with the characterisic intrinsic arginine finger [27] in comparison to a subfamily of 21 other putative small GTPases that lack it (Figure S3). We chose the subfamily of 113 sequences as our MglA set. In contrast, only 63 putative MglB homologs were collected by BLAST analysis, most of which are encoded near members of the MglA set. We used the core regions of the MglB homologs as BLASTP queries to identify more putative MglB partners of our MglA set. The collected sequences were aligned using MAFFT and the core regions were extracted and used to build a phylogenetic tree with FastTree (Figure S4). The tree of 86 putative MglB homologs revealed a subfamily of 71 sequences that were associated with our MglA sequence set based on genome context, and the members of this subfamily were chosen as our final MglB set. MglA and MglB sequences are listed in Table S3.

### Identification of RomR sequences

Initial BLASTP queries with the RomR sequence from *M. xanthus* (MXAN_4461) revealed it to be a multi-domain protein with two regions of conservation, an N-terminal receiver domain and a C-terminal domain that is not homologous to previously characterized domains. Given the ubiquity of receiver domains, we chose to use the C-terminal domain (369–420 of MXAN_4461) in a jackHMMER query against our genome set, which converged after three rounds. The results identified 28 significant hits, 27 of which have N-terminal receiver domains typical of response regulators. We extracted the receiver domain and C-terminal domains of the 27 response regulators sequences (Table S3) and used them as BLASTP queries against our database to identify potential divergent homologs. The queries with the C-terminal regions did not identify any new homologs, whereas the queries with the receiver domains identified 3599 homologs using our default gathering thresholds. Given this large data set, we chose to only gather hits of 1e-20 or lower from the BLASTP queries as this resulted in a set of only 133 sequences, which was more comparable to our previously defined MglA and MglB data sets. The 133 sequences were aligned using the e-ins-i algorithm of MAFFT. We used FastTree to build a phylogenetic tree from the receiver domain regions of the sequences because the remaining portions of the sequences could not be aligned. The resulting tree revealed a subfamily of 31 sequences most of which contain the previously defined C-terminal domain (Figure S5). Those lacking the domain were encoded in genomes from species closely related to their most similar sequence (e.g. two RomR sequences in members of Acidobacteria that lack the C-terminal domain group with a complete RomR sequence from another member of Acidobacteria), which supports their classification as RomR sequences. We chose these 31 sequences for our final RomR set. RomR sequences are listed in Table S3.

### Identification of Frz systems

The Frz system was previously identified as a member of the ACF class of chemosensory systems [61]. We collected the core regions of all the CheA sequences from those analyses and built multiple sequence alignments for each class using MAFFT. Hidden markov models (HMMs) were built from each class specific alignment after being reduced such that no members of the alignment shared more than 80% identity. CheA sequences can be identified by the presence of HATPase_c and CheW domains from Pfam [62], and all sequences with HATPase_c and CheW domains were collected from our genome set. The sequences were compared to our CheA HMM library and assigned to classes based on the highest scoring model. All CheA sequences assigned to the ACF class were collected (164 sequences) and aligned using the e-ins-i algorithm of MAFFT. The core regions corresponding to the P3–P5 domains and the C-terminal receiver domain characteristic of this family were used to build a phylogenetic tree in PhyML. Sequences lacking any of these four domains or the N-terminal histidine phosphotransfer domain were predicted to be non-functional and removed from the analysis. We identified a FrzE specific subfamily in the tree based on Frz system features, genome context, and paralogy events (Figure S6). All FrzE sequences have a FrzZ encoded in nearby genes based on BLASTP queries using neighboring response regulator protein sequences. FrzE sequences are listed in Table S3.

### Identification of gliding motility systems

Recent computational analysis of FAC proteins identified two distinct groups of genes: Group A genes that are only present in organisms that have gliding motility (members of Myxococcales and Bdellovibrionales), and Group B genes that have homologs in the Group A lineages in addition to *Fibrobacter succinogenes* and members of β/γ-proteobacteria for which gliding motility has not been observed [24]. We chose the Group A gene *gltF* as a marker for the presence of gliding motility because it is the most unique based on initial BLAST searches (many Group A genes are putative outer membranes proteins or proteins that contain TPR repeats, both of which result in non-specific BLAST hits). We used the MXAN_4868 GltF sequence as a query in a JackHMMER search, which identified 29 homologs that were present in all Myxococcales and Bdellovibrionales genomes consistent with previous observations [24]. All identified GltF sequences are listed in Table S3.

### Identification of T4P systems

We used the retraction ATPase PilT as a marker for the presence of T4P. The PilT sequences from *M. xanthus* [63], *Neisseria meningitidis* [64], *Pseudomonas aeruginosa* [65], and *Synechocystis* sp. PCC6803 [66] share the same Pfam domain architecture, a single T2SE domain. We collected 3756 sequences from our genome set that matched this domain architecture, aligned them in MAFFT using default settings, and a phylogenetic tree was built from the alignment using FastTree (Figure S7A). This sequence set is expected to also include sequences for PilB and ATPases in type II secretion systems. To identify the branches corresponding to PilT, the PilT sequences from the four aforementioned organisms were used to identify a smaller set of 1219 PilT candidates. The 1219 sequences were realigned in MAFFT using default regions and the core region of the alignment corresponding to residues 5–327 of the *M. xanthus* PilT (MXAN_5787) was extracted and used to build a phylogenetic tree in FastTree (Figure S7B). Identification of characterized PilT proteins in this tree was used to identify a set of 547 PilT sequences (Table S3).

## Supporting Information

Figure S1 Immunoblots of RomR-GFP proteins. Cells were grown as in liquid culture, harvested, and total protein (1 mg per lane) was separated by SDS–PAGE and analyzed by immunoblotting using α-GFP and α-RomR. The different RomR and RomR-GFP proteins are indicated. The migration of molecular size markers is indicated on the left.
(EPS)

Figure S2 Immunoblots of MglB-mCherry accumulation. Cells were grown in liquid culture and harvested, and total protein (1 mg per lane) was separated by SDS–PAGE and analyzed by immunoblotting using α-MglB. MglB and MglB-mCherry including calculated molecular masses are indicated. The migration of molecular size markers in kDa is indicated on the left.
(EPS)

Figure S3 Identification of the MglA family of GTPases. The phylogenetic tree was built from a multiple sequence alignment of candidate MglA sequences identified in similarity searches (Materials and Methods). Branches shown in red represent sequences with the intrinsic arginine finger identified in structural studies of MglA [27]. The sequences in the grey region were chosen as our final MglA set (Table S3).
(EPS)

Figure S4 Identification of the MglB family of GTPase activating proteins. A phylogenetic tree was built from a multiple sequence alignment of candidate MglB sequences identified in similarity searches (Materials and Methods). Branches shown in red represent sequences that are encoded adjacent to the previously identified MglA sequences (Figure S3; Table S3). The sequences in the grey region were chosen as our final MglB set (Table S3).
(EPS)

Figure S5 Identification of the RomR subfamily of response regulators. A phylogenetic tree was built from the receiver domain portion of a multiple sequence alignment of candidate RomR sequences that were identified in similarity searches (Materials and Methods). Branches shown in red represent sequences with the RomR-C domain. The sequences in the grey region were chosen as our final RomR set (Table S3).
(EPS)

Figure S6 Identification of the FrzE subfamily of CheA sequences. A phylogenetic tree was built from a multiple sequence alignment of ACF class CheA sequences (Materials and Methods). Sequence labels are as described in Table S3. FrzE orthologs are readily identifiable in close relatives in of *M. xanthus* (*M. fulvus*, *Corallococcus coralloides*, *Stigmatella aurantiaca*, and four species of *Anaeromyxobacter*). The Che3 system of *M. xanthus* also shows strong similarity to the Frz system based on phylogenetic analysis and gene order conservation. The shown Che systems of *Haliangium ochraceum* (Ha.och) and *Sorangium cellulosum* (So.cel) are similar to both the Frz and Che3 systems. They were assigned to the Frz system because similarity searches with FrzZ identified response regulators encoded in their gene neighborhoods; however, the two systems show differences in gene order and their chemoreceptors are predicted to be membrane bound, unlike the FrzCD receptor. Furthermore, the putative Frz system of *S. cellulosum* lacks essential components (FrzF and FrzG) and may be non-functional. Regardless, the tree reveals that the Frz system is only a very small subfamily of ACF chemotaxis systems.
(EPS)

Figure S7 Identification of the PilT subfamily of Tfp ATPases. (A) A phylogenetic tree was built from an alignment of 3756 proteins that contained the T2SE Pfam domain and only that domain. Branches corresponding to four diverse, experimentally characterized PilT sequences were located in the tree to identify putative PilT sequences: *M. xanthus* (Mx), MXAN_5787; *Neisseria meningitidis* FAM18 (Nm), NMC0036; *Pseudomonas aeruginosa* PAO1 (Pa), PA0395; and *Synechocystis* sp. PCC 6803 (Sy), slr0161. (B) A phylogenetic tree was built from the alignment of the 1219 sequences identified in (A), and the experimentally characterized sequences were located and used to identify a final set of 547 putative PilT sequences (Table S3).
(EPS)

Table S1 Plasmids used in this work.
(DOC)

Table S2 Primers used in this work.
(DOC)

Table S3 MglA, MglB, RomR, FrzE, GltF, and PilT sequences identified in this study.
(DOC)

Text S1 Supplementary Materials and Methods.
(DOC)
