# Supplementary material for: A Response Regulator Interfaces between the Frz Chemosensory System and the MglA/MglB GTPase/GAP Module to Regulate Polarity in Myxococcus xanthus
Source: PLoS Genet. 2012 Sep 13;8(9):e1002951. doi: 10.1371/journal.pgen.1002951 (PMC3441718; doi:10.1371/journal.pgen.1002951)
Supplement: Table S1 — Plasmids used in this work. (DOC) [file pgen.1002951.s008.doc]

**Table S1.** Plasmids used in this study

| **Plasmid** | **Description** | **Source** |
| --- | --- | --- |
| pGFy177 | *PpilA-romR-GFP* in pSWU30 |  |
| pGFy178 | *PpilA-romR*^D53N^*-GFP* in pSWU30 |  |
| pGFy166 | *PpilA-romR*^D53E^*-GFP* in pSWU30 |  |
| pSH1202 | *PpilA-romR*^116-420^*-GFP* |  |
| pDK3 | *PpilA-romR*^369-420^*-GFP* | This work |
| pDK4 | *PpilA-romR*^116-368^*-GFP* | This work |
| pDK5 | *PpilA-romR*^332-420^*-GFP* | This work |
| pDK6 | *PpilA-romR*^116-420^*-GFP* | This work |
| pSL60 | *PpilA-yfp-mglA* in pSW105 |  |
| pTS10 | *PpilA-yfp-mglA*^Q82A^ in pSW105 |  |
| pBJ114 | Vector for generation of in-frame deletions and for gene replacements at native site |  |
| pSL37 | pBJ114 with in-frame deletion cassette for *romR* | This work |
| pFD1 | pBJ114 with in-frame deletion cassette for *frzZ* | This work |
| pDK78 | pBJ114 for integration of *mglB-mCherry* at native site | This work |
| pDK79 | pBJ114 for integration of *mglB-mCherry* at native site and deletion cassette for *mglA* | This work |
| pTS08 | pBJ114 for construction of *mglA*^Q82A^ at native site | This work |
| pGEX4T | Vector for GST overexpression | GE-Healthcare |
| pSL54 | For GST-MglA overexpression in pGEX4T |  |
| pMal-c2 | Vector for MalE overexpression | New England Biolabs |
| pDK28 | For MalE-RomR overexpression in pMal-c2 | This work |
| pET45 | For overexpression of His_6_-tagged protein | This work |
| pDK47 | For His_6_-RomR overexpression in pET45 | This work |
| pES1 | For His_6_-MglB overexpression in pET45 | This work |
| MglA-His_6_ | For MglA-His_6_ overexpression |  |
| pBlueskript II SK- | cloning vector | Fermentas |

**References**

1. Leonardy S, Freymark G, Hebener S, Ellehauge E, Søgaard-Andersen L (2007) Coupling of protein localization and cell movements by a dynamically localized response regulator in *Myxococcus xanthus*. EMBO J 26: 4433–4444.

2. Leonardy S, Miertzschke M, Bulyha I, Sperling E, Wittinghofer A, et al. (2010) Regulation of dynamic polarity switching in bacteria by a Ras-like G-protein and its cognate GAP. EMBO J 29: 2276-2289.

3. Miertzschke M, Koerner C, Vetter IR, Keilberg D, Hot E, et al. (2011) Structural analysis of the Ras-like G protein MglA and its cognate GAP MglB and implications for bacterial polarity. EMBO J 30: 4185-4197.

4. Julien B, Kaiser AD, Garza A (2000) Spatial control of cell differentiation in *Myxococcus xanthus*. Proc Natl Acad Sci USA 97: 9098-9103.

5. Zhang Y, Franco M, Ducret A, Mignot T (2010) A bacterial Ras-like small GTP-binding protein and its cognate GAP establish a dynamic spatial polarity axis to control directed motility. PLoS Biol 8: e1000430.
