# Supplementary material for: A Response Regulator Interfaces between the Frz Chemosensory System and the MglA/MglB GTPase/GAP Module to Regulate Polarity in Myxococcus xanthus
Source: PLoS Genet. 2012 Sep 13;8(9):e1002951. doi: 10.1371/journal.pgen.1002951 (PMC3441718; doi:10.1371/journal.pgen.1002951)
Supplement: Table S2 — Primers used in this work. (DOC) [file pgen.1002951.s009.doc]

**Table S2.** Primers used in this work

| **Name** | **Sequence (5’-3’)** |
| --- | --- |
| oMglA-EcoRI | ATCCGGAATTCATGTCCTTCATCAATTAC |
| oMglAstop-NotI | ATCGCGGCGGCCGCCTCAAGAAGGGTGGTTGA |
| oDromR-1 | ATCGGTCTAGACATCGCGGAGGCGCTGCC |
| oDromR-2 | GAGCTCCTCGCGGATGGTGAGCGAGTC |
| oDromR-3 | ACCATCCGCGAGGAGCTCGAGCGGCTC |
| oDromR-4 | ATCGGAAGCTTCTCGCGCACCGCGGCGGA |
| oMglAQ82Aforw | ACGGTGCCCGGTGCAGTCTTCTACGAC |
| oMglAQ82Arev | GTCGTAGAAGACTGCACCGGGCACCGT |
| omglB3 | ATCCGGATCCGATGGGCACGCAACTGGTG |
| omglB4 | ATCGGGAATTCCCTTGAGCGTGTCGAAGA |
| HisRomRPstI | atcggCTGCAGATGCCCAAGAATCTGCTGGTCGC |
| HisRomRrv | atcggAAGCTTTCAGTGCTGGGTCTCTCGGTCCTTGA |
| MalE-RomRfw | atcggGAATTCATGCCCAAGAATCTGCTGGTCGC |
| MalE-RomRrv | atcggAAGCTTTCAGTGCTGGGTCTCTCGGTCC |
| MglBfwsur | atcggaagcttGCGTGAAGCCCTCATAGGTGAGC |
| MglBrvmcherry | gctcaccatCTCGCTGAAGAGGTTGTCGATATCG |
| Mcherryfw | ttcagcgagATGGTGAGCAAGGGCGAGGAGGAT |
| Mcherryrv | cttcccgggTTACTTGTACAGCTCGTCCATGCCG |
| MglAfw | tacaagtaaCCCGGGAAGCCATGTCCTTC |
| MglAsurrv | atcggGAATTCACGGGTGACGGGCGGCGGGG |
| FrzZA | atcggGAATTCAGCTGCCCGTGACGCCGACGAA |
| FrzZB | CAGCTCCTTGGCGCTGTCATCAATGACCAGTA |
| FrzZC | TTGATGACAGCGCCAAGGAGCTGATGCCCACC |
| FrzZD | atcggAAGCTTCCCTCTTCGACGCGGGGCTG |
| DA1 | atcggTCTAGAATGAAGGCGCTGGTCGGC |
| DA2 | atcggGATATCAGGCGCACGGGCGCTCGC |
| DA3 | atcggTCTAGAATGGCCGCGGATGGGGGC |
| DA4 | atcggGATATCGTGCTGGGTCTCTCGGTC |
| DA5 | ATCGGTCTAGAATGTCCATCAGCATCGAGGA |
| oCrGFP-3 | atcggGATATCATGGCCAAGGGCGAGGAG |
| oCrGFP-2 | atcggAAGCTTTTACTTGTACAGCTCGTCCATGCC |
