# Supplementary material for: A Response Regulator Interfaces between the Frz Chemosensory System and the MglA/MglB GTPase/GAP Module to Regulate Polarity in Myxococcus xanthus
Source: PLoS Genet. 2012 Sep 13;8(9):e1002951. doi: 10.1371/journal.pgen.1002951 (PMC3441718; doi:10.1371/journal.pgen.1002951)
Supplement: Table S3 — MglA, MglB, RomR, FrzE, GltF, and PilT sequences identified in this study. (DOC) [file pgen.1002951.s010.doc]

| **Table S3. MglA, MglB, RomR, FrzE, GltF, and PilT sequences identified in this study*** | | | | |  |  |
| --- | --- | --- | --- | --- | --- | --- |
| **Genome name and Project ID (uid)** | **MglA** | **MglB** | **RomR** | **FrzE** | **GltF** | **PilT** |
| Acaryochloris_marina_MBIC11017_uid58167 |  |  |  |  |  | AM1_0418 |
| Acetobacterium_woodii_DSM_1030_uid88073 |  |  |  |  |  | Awo_c01990 |
| Acetohalobium_arabaticum_DSM_5501_uid51423 |  |  |  |  |  | Acear_1773 |
| Acidithiobacillus_caldus_SM_1_uid70791 |  |  |  |  |  | Atc_2605 |
| Acidithiobacillus_ferrivorans_SS3_uid67387 |  |  |  |  |  | Acife_2763 |
| Acidithiobacillus_ferrooxidans_ATCC_23270_uid57649 |  |  |  |  |  | AFE_0264 |
| Acidithiobacillus_ferrooxidans_ATCC_53993_uid58613 |  |  |  |  |  | Lferr_0441 |
| Acidothermus_cellulolyticus_11B_uid58501 |  |  |  |  |  | Acel_1321 |
| Acidovorax_avenae_ATCC_19860_uid42497 |  |  |  |  |  | Acav_0607 |
| Acidovorax_citrulli_AAC00_1_uid58429 |  |  |  |  |  | Aave_0637 |
| Acidovorax_ebreus_TPSY_uid59233 |  |  |  |  |  | Dtpsy_0412 |
| Acidovorax_JS42_uid58427 |  |  |  |  |  | Ajs_0420 |
| Acinetobacter_ADP1_uid61597 |  |  |  |  |  | ACIAD0912 |
| Acinetobacter_baumannii_AB0057_uid59083 |  |  |  |  |  | AB57_0945 |
| Acinetobacter_baumannii_AB307_0294_uid59271 |  |  |  |  |  | ABBFA_002717 |
| Acinetobacter_baumannii_ACICU_uid58765 |  |  |  |  |  | ACICU_00848 |
| Acinetobacter_baumannii_ATCC_17978_uid58731 |  |  |  |  |  | A1S_0897 |
| Acinetobacter_baumannii_AYE_uid61637 |  |  |  |  |  | ABAYE2918 |
| Acinetobacter_calcoaceticus_PHEA_2_uid83123 |  |  |  |  |  | BDGL_000161 |
| Acinetobacter_oleivorans_DR1_uid50119 |  |  |  |  |  | AOLE_15225 |
| Aeromonas_hydrophila_ATCC_7966_uid58617 |  |  |  |  |  | AHA_3665 |
| Aeromonas_salmonicida_A449_uid58631 |  |  |  |  |  | ASA_3632 |
| Aeromonas_veronii_B565_uid66323 |  |  |  |  |  | B565_3513 |
| Akkermansia_muciniphila_ATCC_BAA_835_uid58985 |  |  |  |  |  | Amuc_0165, Amuc_0166 |
| Alcanivorax_borkumensis_SK2_uid58169 |  |  |  |  |  | ABO_2670 |
| Alicycliphilus_denitrificans_BC_uid49953 |  |  |  |  |  | Alide_0493 |
| Alicycliphilus_denitrificans_K601_uid66307 |  |  |  |  |  | Alide2_0450 |
| Alicyclobacillus_acidocaldarius_DSM_446_uid59199 |  |  |  |  |  | Aaci_0310 |
| Aliivibrio_salmonicida_LFI1238_uid59251 |  |  |  |  |  | VSAL_I0544 |
| Alkalilimnicola_ehrlichii_MLHE_1_uid58467 |  |  |  |  |  | Mlg_0342 |
| Alkaliphilus_metalliredigens_QYMF_uid58171 |  |  |  |  |  | Amet_3481 |
| Alkaliphilus_oremlandii_OhILAs_uid58495 |  |  |  |  |  | Clos_1637 |
| Allochromatium_vinosum_DSM_180_uid46083 |  |  |  |  |  | Alvin_2616 |
| Alteromonas_macleodii__Deep_ecotype__uid58251 |  |  |  |  |  | MADE_1015345 |
| Alteromonas_SN2_uid67349 |  |  |  |  |  | ambt_02040 |
| Ammonifex_degensii_KC4_uid41053 |  |  |  |  |  | Adeg_1724 |
| Amycolatopsis_mediterranei_U32_uid50565 |  |  |  |  |  | AMED_4452 |
| Anabaena_variabilis_ATCC_29413_uid58043 |  |  |  |  |  | Ava_0375 |
| Anaeromyxobacter_dehalogenans_2CP_1_uid58989 | A2cp1_1284, A2cp1_3774, A2cp1_4477 | A2cp1_3775 | A2cp1_1568 | A2cp1_0638 | A2cp1_3464 | A2cp1_0657 |
| Anaeromyxobacter_dehalogenans_2CP_C_uid58135 | Adeh_1154, Adeh_3633, Adeh_4322 | Adeh_3634 | Adeh_2391 | Adeh_0613 | Adeh_3320 | Adeh_0623 |
| Anaeromyxobacter_Fw109_5_uid58755 | Anae109_1201, Anae109_3758, Anae109_4467 | Anae109_3759 | Anae109_1476 | Anae109_0657 | Anae109_3384 | Anae109_0668 |
| Anaeromyxobacter_K_uid58953 | AnaeK_1214, AnaeK_3691, AnaeK_4458 | AnaeK_3692 | AnaeK_1473 | AnaeK_0647 | AnaeK_3400 | AnaeK_0657 |
| Anoxybacillus_flavithermus_WK1_uid59135 |  |  |  |  |  | Aflv_0625 |
| Aquifex_aeolicus_VF5_uid57765 | aq_1560, aq_1823 | aq_1822 |  |  |  | aq_745 |
| Aromatoleum_aromaticum_EbN1_uid58231 |  |  |  |  |  | ebA1770 |
| Azoarcus_BH72_uid61603 |  |  |  |  |  | azo3468 |
| Azotobacter_vinelandii_DJ_uid57597 |  |  |  |  |  | Avin_03090 |
| Bacillus_cellulosilyticus_DSM_2522_uid43329 |  |  |  |  |  | Bcell_3102 |
| Bacillus_coagulans_2_6_uid68053 |  |  |  |  |  | BCO26_1869 |
| Bacillus_coagulans_36D1_uid54335 |  |  |  |  |  | Bcoa_2631 |
| Bacillus_pseudofirmus_OF4_uid45847 |  |  |  |  |  | BpOF4_02870 |
| Bacillus_selenitireducens_MLS10_uid49513 |  |  |  |  |  | Bsel_1440 |
| Bacteriovorax_marinus_SJ_uid82341 | BMS_0054 |  | BMS_3223 |  | BMS_0266, BMS_1383 | BMS_0120 |
| Bdellovibrio_bacteriovorus_HD100_uid61595 | Bd3734 |  | Bd2761 |  | Bd1477, Bd2373, Bd3600 | Bd3852 |
| Bordetella_bronchiseptica_RB50_uid57613 |  |  |  |  |  | BB0791 |
| Butyrivibrio_proteoclasticus_B316_uid51489 |  |  |  |  |  | bpr_I2013 |
| Caldicellulosiruptor_bescii_DSM_6725_uid59201 |  |  |  |  |  | Athe_1883 |
| Caldicellulosiruptor_hydrothermalis_108_uid60157 |  |  |  |  |  | Calhy_0897 |
| Caldicellulosiruptor_kristjanssonii_177R1B_uid60393 |  |  |  |  |  | Calkr_0814 |
| Caldicellulosiruptor_kronotskyensis_2002_uid60491 |  |  |  |  |  | Calkro_0832 |
| Caldicellulosiruptor_lactoaceticus_6A_uid60575 |  |  |  |  |  | Calla_1519 |
| Caldicellulosiruptor_obsidiansis_OB47_uid51501 |  |  |  |  |  | COB47_1690 |
| Caldicellulosiruptor_owensensis_OL_uid60165 |  |  |  |  |  | Calow_1602 |
| Caldicellulosiruptor_saccharolyticus_DSM_8903_uid58289 |  |  |  |  |  | Csac_1061 |
| Calditerrivibrio_nitroreducens_DSM_19672_uid60821 | Calni_1376 | Calni_1375 | Calni_0658 |  |  | Calni_1207,  Calni_1647 |
| Campylobacter_curvus_525_92_uid58669 |  |  |  |  |  | CCV52592_0998 |
| Campylobacter_fetus_82_40_uid58545 |  |  |  |  |  | CFF8240_1145 |
| Campylobacter_hominis_ATCC_BAA_381_uid58981 |  |  |  |  |  | CHAB381_1643 |
| Candidatus_Accumulibacter_phosphatis_clade_IIA_UW_1_uid59207 |  |  |  |  |  | CAP2UW1_3601 |
| Candidatus_Chloracidobacterium_thermophilum_B_uid73587 | Cabther_A0781 | Cabther_A0780 | Cabther_A0541 |  |  | Cabther_A1368, Cabther_A1697 |
| Candidatus_Cloacamonas_acidaminovorans_uid62959 |  |  |  |  |  | CLOAM1400 |
| Candidatus_Desulforudis_audaxviator_MP104C_uid59067 |  |  |  |  |  | Daud_0951, Daud_0969 |
| Candidatus_Koribacter_versatilis_Ellin345_uid58479 | Acid345_0708 |  | Acid345_0072 |  |  | Acid345_1391 |
| Candidatus_Nitrospira_defluvii_uid51175 |  |  |  |  |  | NIDE3448 |
| Candidatus_Solibacter_usitatus_Ellin6076_uid58139 |  |  | Acid_3193 |  |  | Acid_1587 |
| Carboxydothermus_hydrogenoformans_Z_2901_uid57821 |  |  |  |  |  | CHY_0631 |
| Cellulomonas_fimi_ATCC_484_uid66779 |  |  |  |  |  | Celf_2006 |
| Cellulomonas_flavigena_DSM_20109_uid48821 |  |  |  |  |  | Cfla_1823 |
| Cellvibrio_gilvus_ATCC_13127_uid68143 |  |  |  |  |  | Celgi_1812 |
| Cellvibrio_japonicus_Ueda107_uid59139 |  |  |  |  |  | CJA_0087 |
| Chloroflexus_aggregans_DSM_9485_uid58621 | Cagg_2679 | Cagg_0992, Cagg_2680, Cagg_3071 |  |  |  |  |
| Chloroflexus_aurantiacus_J_10_fl_uid57657 | Caur_2060 | Caur_2061, Caur_2319, Caur_3048 |  |  |  |  |
| Chloroflexus_Y_400_fl_uid59085 | Chy400_2221 | Chy400_2222, Chy400_2499, Chy400_3294 |  |  |  |  |
| Chloroherpeton_thalassium_ATCC_35110_uid59187 | Ctha_0400, Ctha_2360 | Ctha_0401, Ctha_2351 |  |  |  |  |
| Chromobacterium_violaceum_ATCC_12472_uid58001 |  |  |  |  |  | CV_0179 |
| Chromohalobacter_salexigens_DSM_3043_uid62921 |  |  |  |  |  | Csal_3053 |
| Clostridium_acetobutylicum_ATCC_824_uid57677 |  |  |  |  |  | CA_C1690 |
| Clostridium_acetobutylicum_DSM_1731_uid68293 |  |  |  |  |  | SMB_G1715 |
| Clostridium_beijerinckii_NCIMB_8052_uid58137 |  |  |  |  |  | Cbei_1116 |
| Clostridium_BNL1100_uid84307 |  |  |  |  |  | Clo1100_0837 |
| Clostridium_botulinum_A_ATCC_19397_uid58927 |  |  |  |  |  | CLB_2415 |
| Clostridium_botulinum_A_ATCC_3502_uid61579 |  |  |  |  |  | CBO2539 |
| Clostridium_botulinum_A_Hall_uid58931 |  |  |  |  |  | CLC_2397 |
| Clostridium_botulinum_A2_Kyoto_uid59229 |  |  |  |  |  | CLM_2843 |
| Clostridium_botulinum_A3_Loch_Maree_uid59149 |  |  |  |  |  | CLK_1922 |
| Clostridium_botulinum_B_Eklund_17B_uid59159 |  |  |  |  |  | CLL_A1186 |
| Clostridium_botulinum_B1_Okra_uid59147 |  |  |  |  |  | CLD_2097 |
| Clostridium_botulinum_Ba4_657_uid59173 |  |  |  |  |  | CLJ_B2769 |
| Clostridium_botulinum_BKT015925_uid66203 |  |  |  |  |  | CbC4_1532 |
| Clostridium_botulinum_E3_Alaska_E43_uid59157 |  |  |  |  |  | CLH_1137 |
| Clostridium_botulinum_F_Langeland_uid58929 |  |  |  |  |  | CLI_2601 |
| Clostridium_cellulolyticum_H10_uid58709 |  |  |  |  |  | Ccel_2413 |
| Clostridium_cellulovorans_743B_uid51503 |  |  |  |  |  | Clocel_1842 |
| Clostridium_clariflavum_DSM_19732_uid82345 |  |  |  |  |  | Clocl_1391,  Clocl_3324 |
| Clostridium_difficile_630_uid57679 |  |  |  |  |  | CD3505 |
| Clostridium_difficile_CD196_uid41017 |  |  |  |  |  | CD196_3296 |
| Clostridium_difficile_R20291_uid40921 |  |  |  |  |  | CDR20291_3342 |
| Clostridium_kluyveri_DSM_555_uid58885 |  |  |  |  |  | CKL_1336 |
| Clostridium_kluyveri_NBRC_12016_uid59369 |  |  |  |  |  | CKR_1232 |
| Clostridium_lentocellum_DSM_5427_uid49117 |  |  |  |  |  | Clole_1717 |
| Clostridium_ljungdahlii_DSM_13528_uid50583 |  |  |  |  |  | CLJU_c12270 |
| Clostridium_novyi_NT_uid58643 |  |  |  |  |  | NT01CX_2266 |
| Clostridium_perfringens_13_uid57681 |  |  |  |  |  | CPE1767 |
| Clostridium_perfringens_ATCC_13124_uid57901 |  |  |  |  |  | CPF_2020 |
| Clostridium_perfringens_SM101_uid58117 |  |  |  |  |  | CPR_1737 |
| Clostridium_saccharolyticum_WM1_uid51419 |  |  |  |  |  | Closa_1305 |
| Clostridium_sticklandii_DSM_519_uid59585 |  |  |  |  |  | CLOST_0185, CLOST_0861 |
| Clostridium_tetani_E88_uid57683 |  |  |  |  |  | CTC01119 |
| Clostridium_thermocellum_ATCC_27405_uid57917 |  |  |  |  |  | Cthe_0257,  Cthe_1106 |
| Collimonas_fungivorans_Ter331_uid70793 |  |  |  |  |  | CFU_0755 |
| Colwellia_psychrerythraea_34H_uid57855 |  |  |  |  |  | CPS_3662 |
| Comamonas_testosteroni_CNB_2_uid62961 |  |  |  |  |  | CtCNB1_4408 |
| Coprothermobacter_proteolyticus_DSM_5265_uid59253 |  |  |  |  |  | COPRO5265_0771 |
| Coraliomargarita_akajimensis_DSM_45221_uid47079 |  |  |  |  |  | Caka_1448 |
| Corallococcus_coralloides_DSM_2259_uid82779 | COCOR_01949, COCOR_05345, COCOR_07291 | COCOR_01950 | COCOR_03318 | COCOR_03835 | COCOR_01216, COCOR_01944, COCOR_02561, COCOR_03240 | COCOR_06287, COCOR_07293, COCOR_07294 |
| Cupriavidus_metallidurans_CH34_uid57815 |  |  |  |  |  | Rmet_2936 |
| Cupriavidus_necator_N_1_uid68689 |  |  |  |  |  | CNE_1c30600 |
| Cupriavidus_taiwanensis_LMG_19424_uid61615 |  |  |  |  |  | RALTA_A2579 |
| Cyanothece_ATCC_51142_uid59013 |  |  |  |  |  | cce_0419 |
| Cyanothece_PCC_7424_uid59025 |  |  |  |  |  | PCC7424_4183 |
| Cyanothece_PCC_7425_uid59435 |  |  |  |  |  | Cyan7425_4696 |
| Cyanothece_PCC_7822_uid52547 |  |  |  |  |  | Cyan7822_1803 |
| Cyanothece_PCC_8801_uid59027 |  |  |  |  |  | PCC8801_1611 |
| Cyanothece_PCC_8802_uid59143 |  |  |  |  |  | Cyan8802_1637 |
| Dechloromonas_aromatica_RCB_uid58025 | Daro_0968 |  |  |  |  | Daro_3883 |
| Dechlorosoma_suillum_PS_uid81439 |  |  |  |  |  | Dsui_0604 |
| Deferribacter_desulfuricans_SSM1_uid46653 | DEFDS_0024 | DEFDS_0025 | DEFDS_0166 |  |  | DEFDS_0625, DEFDS_1110 |
| Dehalococcoides_BAV1_uid58477 |  |  |  |  |  | DehaBAV1_0806, DehaBAV1_1066, DehaBAV1_1171 |
| Dehalococcoides_CBDB1_uid58413 |  |  |  |  |  | cbdb_A1178, cbdb_A1310, cbdb_A873 |
| Dehalococcoides_ethenogenes_195_uid57763 |  |  |  |  |  | DET0922,  DET1255,  DET1359 |
| Dehalococcoides_GT_uid42115 |  |  |  |  |  | DehalGT_0767, DehalGT_0992 |
| Dehalococcoides_VS_uid42393 |  |  |  |  |  | DhcVS_1038, DhcVS_1141, DhcVS_793 |
| Dehalogenimonas_lykanthroporepellens_BL_DC_9_uid48131 |  |  |  |  |  | Dehly_0313, Dehly_0787, Dehly_0905, Dehly_1228 |
| Deinococcus_deserti_VCD115_uid58615 | Deide_17750 | Deide_17760 |  |  |  | Deide_19600 |
| Deinococcus_geothermalis_DSM_11300_uid58275 | Dgeo_1815 | Dgeo_1816 |  |  |  | Dgeo_0264 |
| Deinococcus_maricopensis_DSM_21211_uid62225 | Deima_2991 | Deima_2990 |  |  |  | Deima_0021 |
| Deinococcus_proteolyticus_MRP_uid63399 | Deipr_0325 | Deipr_0324 |  |  |  | Deipr_1313, Deipr_2450 |
| Deinococcus_radiodurans_R1_uid57665 | DR_0853 | DR_0854 |  |  |  | DR_1963 |
| Delftia_acidovorans_SPH_1_uid58703 |  |  |  |  |  | Daci_1068 |
| Delftia_Cs1_4_uid67319 |  |  |  |  |  | DelCs14_5453 |
| Denitrovibrio_acetiphilus_DSM_12809_uid46657 |  |  | Dacet_3018 |  |  | Dacet_1334 |
| Desulfatibacillum_alkenivorans_AK_01_uid58913 | Dalk_0302, Dalk_2485 | Dalk_2486 |  |  |  | Dalk_3302,  Dalk_3612,  Dalk_3613 |
| Desulfitobacterium_hafniense_DCB_2_uid57749 |  |  |  |  |  | Dhaf_3527 |
| Desulfitobacterium_hafniense_Y51_uid58605 |  |  |  |  |  | DSY2385 |
| Desulfobacterium_autotrophicum_HRM2_uid59061 | HRM2_35580 | HRM2_35590 |  |  |  | HRM2_04990 |
| Desulfobulbus_propionicus_DSM_2032_uid62265 | Despr_1663 | Despr_1662 |  |  |  | Despr_2329 |
| Desulfococcus_oleovorans_Hxd3_uid58777 | Dole_2372 |  |  |  |  | Dole_3053 |
| Desulfohalobium_retbaense_DSM_5692_uid59183 |  |  |  |  |  | Dret_1917 |
| Desulfomicrobium_baculatum_DSM_4028_uid59217 |  |  |  |  |  | Dbac_1131 |
| Desulfosporosinus_orientis_DSM_765_uid82939 |  |  |  |  |  | Desor_1011 |
| Desulfotalea_psychrophila_LSv54_uid58153 | DP2776 | DP2777 |  |  |  | DP1694 |
| Desulfotomaculum_acetoxidans_DSM_771_uid59109 |  |  |  |  |  | Dtox_2666, Dtox_3244,  Dtox_3256 |
| Desulfotomaculum_carboxydivorans_CO_1_SRB_uid67317 |  |  |  |  |  | Desca_2182 |
| Desulfotomaculum_kuznetsovii_DSM_6115_uid67357 |  |  |  |  |  | Desku_1984 |
| Desulfotomaculum_reducens_MI_1_uid58277 |  |  |  |  |  | Dred_1035 |
| Desulfotomaculum_ruminis_DSM_2154_uid67507 |  |  |  |  |  | Desru_0464, Desru_3111 |
| Desulfovibrio_africanus_Walvis_Bay_uid66847 |  |  |  |  |  | Desaf_0885 |
| Desulfovibrio_magneticus_RS_1_uid59309 |  |  |  |  |  | DMR_06690 |
| Desulfovibrio_vulgaris__Miyazaki_F__uid59089 |  |  |  |  |  | DvMF_1038 |
| Desulfovibrio_vulgaris_DP4_uid58679 |  |  |  |  |  | Dvul_1802 |
| Desulfovibrio_vulgaris_Hildenborough_uid57645 |  |  |  |  |  | DVU1262 |
| Desulfurispirillum_indicum_S5_uid45897 |  |  |  |  |  | Selin_2217 |
| Desulfurivibrio_alkaliphilus_AHT2_uid49487 |  |  |  |  |  | DaAHT2_1068 |
| Desulfurobacterium_thermolithotrophum_DSM_11699_uid63405 | Dester_1068 | Dester_1069 | Dester_0564 |  |  | Dester_0294 |
| Dichelobacter_nodosus_VCS1703A_uid57643 |  |  |  |  |  | DNO_0675 |
| Dictyoglomus_thermophilum_H_6_12_uid59439 | DICTH_1242 | DICTH_1243 |  |  |  | DICTH_0722 |
| Dictyoglomus_turgidum_DSM_6724_uid59177 | Dtur_1356 | Dtur_1357 |  |  |  | Dtur_0874 |
| Eggerthella_lenta_DSM_2243_uid59079 |  |  |  |  |  | Elen_0043,  Elen_0097 |
| Eggerthella_YY7918_uid68707 |  |  |  |  |  | EGYY_01180, EGYY_27870 |
| Ethanoligenens_harbinense_YUAN_3_uid46255 |  |  |  |  |  | Ethha_0792 |
| Eubacterium_eligens_ATCC_27750_uid59171 |  |  |  |  |  | EUBELI_00785, EUBELI_01703 |
| Eubacterium_limosum_KIST612_uid59777 |  |  |  |  |  | ELI_0445,  ELI_4149 |
| Eubacterium_rectale_ATCC_33656_uid59169 |  |  |  |  |  | EUBREC_1529, EUBREC_2195 |
| Exiguobacterium_AT1b_uid59093 |  |  |  |  |  | EAT1b_2661 |
| Exiguobacterium_sibiricum_255_15_uid58053 |  |  |  |  |  | Exig_2123 |
| Ferrimonas_balearica_DSM_9799_uid53371 |  |  |  |  |  | Fbal_2942 |
| Fervidobacterium_nodosum_Rt17_B1_uid58625 |  |  |  |  |  | Fnod_0278 |
| Fibrobacter_succinogenes_S85_uid41169 | Fisuc_0227 | Fisuc_0226 |  |  |  | Fisuc_1051 |
| Filifactor_alocis_ATCC_35896_uid46625 |  |  |  |  |  | HMPREF0389_00425 |
| Flexistipes_sinusarabici_DSM_4947_uid68147 | Flexsi_0290 | Flexsi_0291 | Flexsi_0107 |  |  | Flexsi_1221, Flexsi_1371 |
| Francisella_novicida_U112_uid58499 |  |  |  |  |  | FTN_1622 |
| Francisella_philomiragia_ATCC_25017_uid59105 |  |  |  |  |  | Fphi_0996 |
| Francisella_tularensis_FSC198_uid58693 |  |  |  |  |  | FTF0088 |
| Francisella_tularensis_holarctica_LVS_uid58595 |  |  |  |  |  | FTL_1770 |
| Francisella_tularensis_SCHU_S4_uid57589 |  |  |  |  |  | FTT_0088 |
| Francisella_tularensis_TI0902_uid89373 |  |  |  |  |  | FTV_0084 |
| Francisella_tularensis_TIGB03_uid89379 |  |  |  |  |  | FTU_0084 |
| Francisella_tularensis_WY96_3418_uid58811 |  |  |  |  |  | FTW_0165 |
| Francisella_TX077308_uid68321 |  |  |  |  |  | F7308_0599 |
| Gallionella_capsiferriformans_ES_2_uid51505 |  |  |  |  |  | Galf_2682 |
| gamma_proteobacterium_HdN1_uid51635 |  |  |  |  |  | HDN1F_37260 |
| Gemmatimonas_aurantiaca_T_27_uid58813 | GAU_1116, GAU_1410 | GAU_1115 |  |  |  | GAU_2569,  GAU_3128 |
| Geobacillus_C56_T3_uid49467 |  |  |  |  |  | GC56T3_0863 |
| Geobacillus_kaustophilus_HTA426_uid58227 |  |  |  |  |  | GK2628 |
| Geobacillus_thermodenitrificans_NG80_2_uid58829 |  |  |  |  |  | GTNG_2558 |
| Geobacillus_thermoglucosidasius_C56_YS93_uid48129 |  |  |  |  |  | Geoth_1012 |
| Geobacillus_thermoleovorans_CCB_US3_UF5_uid82949 |  |  |  |  |  | GTCCBUS3UF5_29540 |
| Geobacillus_WCH70_uid59045 |  |  |  |  |  | GWCH70_2561 |
| Geobacillus_Y4_1MC1_uid55779 |  |  |  |  |  | GY4MC1_0942 |
| Geobacillus_Y412MC52_uid55381 |  |  |  |  |  | GYMC52_2664 |
| Geobacillus_Y412MC61_uid41171 |  |  |  |  |  | GYMC61_0889 |
| Geobacter_bemidjiensis_Bem_uid58749 | Gbem_2315, Gbem_3962 | Gbem_3963 | Gbem_1530 |  |  | Gbem_0084, Gbem_2594 |
| Geobacter_FRC_32_uid58543 | Geob_0373, Geob_2389, Geob_3508 | Geob_0374 | Geob_3083 |  |  | Geob_0439, Geob_3373 |
| Geobacter_lovleyi_SZ_uid58713 | Glov_1322, Glov_3124 | Glov_3123 | Glov_2486 |  |  | Glov_1654, Glov_3221 |
| Geobacter_M18_uid55771 | GM18_0303, GM18_1915, GM18_4429 | GM18_4430 | GM18_1365 |  |  | GM18_0227, GM18_2496 |
| Geobacter_M21_uid59037 | GM21_1908, GM21_2366, GM21_4048 | GM21_4049 | GM21_2686 |  |  | GM21_0067, GM21_1632 |
| Geobacter_metallireducens_GS_15_uid57731 | Gmet_1982, Gmet_3417, Gmet_3475 | Gmet_3418 | Gmet_0955 |  |  | Gmet_1394, Gmet_3400 |
| Geobacter_sulfurreducens_PCA_uid57743 | GSU0099, GSU1930 | GSU0098 | GSU2046 |  |  | GSU0436,  GSU1492 |
| Geobacter_uraniireducens_Rf4_uid58475 | Gura_2812, Gura_4332 | Gura_4331 | Gura_1798 |  |  | Gura_2681,  Gura_4274 |
| Glaciecola_4H_3_7_YE_5_uid66595 |  |  |  |  |  | Glaag_3708 |
| Glaciecola_nitratireducens_FR1064_uid73759 |  |  |  |  |  | GNIT_2744 |
| Gloeobacter_violaceus_PCC_7421_uid58011 |  |  |  |  |  | gvip376 |
| Granulicella_mallensis_MP5ACTX8_uid49957 |  |  |  |  |  | AciX8_1088 |
| Hahella_chejuensis_KCTC_2396_uid58483 | HCH_00229 |  |  |  |  | HCH_06352 |
| Halanaerobium_hydrogeniformans_uid60191 |  |  |  |  |  | Halsa_1447 |
| Haliangium_ochraceum_DSM_14365_uid41425 | Hoch_2556, Hoch_6866 | Hoch_6867 | Hoch_3476 | Hoch_6787 | Hoch_0046, Hoch_3963, Hoch_5628 | Hoch_4827 |
| Halomonas_elongata_DSM_2581_uid52781 |  |  |  |  |  | HELO_4113 |
| Halorhodospira_halophila_SL1_uid58473 |  |  |  |  |  | Hhal_0316 |
| Halothermothrix_orenii_H_168_uid58585 |  |  |  |  |  | Hore_05980 |
| Halothiobacillus_neapolitanus_c2_uid41317 |  |  |  |  |  | Hneap_1841 |
| Heliobacterium_modesticaldum_Ice1_uid58279 |  |  |  |  |  | HM1_0250 |
| Herminiimonas_arsenicoxydans_uid58291 |  |  |  |  |  | HEAR0289 |
| Herpetosiphon_aurantiacus_DSM_785_uid58599 | Haur_0931, Haur_1007 | Haur_0932, Haur_1268 |  |  |  |  |
| Hippea_maritima_DSM_10411_uid65267 |  |  |  |  |  | Hipma_0475 |
| Hydrogenobacter_thermophilus_TK_6_uid45927 | HTH_0335, HTH_1432, HTH_1830 | HTH_1831 |  |  |  | HTH_0302 |
| Hydrogenobaculum_Y04AAS1_uid58857 |  |  |  |  |  | HY04AAS1_0245 |
| Idiomarina_loihiensis_L2TR_uid58087 |  |  |  |  |  | IL1973 |
| Ilyobacter_polytropus_DSM_2926_uid59769 |  |  |  |  |  | Ilyop_0248 |
| Isosphaera_pallida_ATCC_43644_uid62207 |  |  |  |  |  | Isop_2820 |
| Janthinobacterium_Marseille_uid58603 |  |  |  |  |  | mma_0340 |
| Jonesia_denitrificans_DSM_20603_uid59053 |  |  |  |  |  | Jden_1346 |
| Kangiella_koreensis_DSM_16069_uid59209 |  |  |  |  |  | Kkor_0218 |
| Kineococcus_radiotolerans_SRS30216_uid58067 |  |  |  |  |  | Krad_3027 |
| Kosmotoga_olearia_TBF_19_5_1_uid59205 |  |  |  |  |  | Kole_0842 |
| Kyrpidia_tusciae_DSM_2912_uid48361 |  |  |  |  |  | Btus_0664 |
| Laribacter_hongkongensis_HLHK9_uid59265 |  |  |  |  |  | LHK_00032 |
| Legionella_longbeachae_NSW150_uid46099 |  |  |  |  |  | LLO_0899 |
| Legionella_pneumophila_2300_99_Alcoy_uid48801 |  |  |  |  |  | lpa_02934 |
| Legionella_pneumophila_ATCC_43290_uid86885 |  |  |  |  |  | lp12_1954 |
| Legionella_pneumophila_Corby_uid58733 |  |  |  |  |  | LPC_1497 |
| Legionella_pneumophila_Lens_uid58209 |  |  |  |  |  | lpl1990 |
| Legionella_pneumophila_Paris_uid58211 |  |  |  |  |  | lpp1995 |
| Legionella_pneumophila_Philadelphia_1_uid57609 |  |  |  |  |  | lpg2013 |
| Leptothrix_cholodnii_SP_6_uid58971 |  |  |  |  |  | Lcho_1631 |
| Magnetococcus_MC_1_uid57833 |  |  |  |  |  | Mmc1_1609 |
| Mahella_australiensis_50_1_BON_uid66917 |  |  |  |  |  | Mahau_1131 |
| Marinithermus_hydrothermalis_DSM_14884_uid65783 | Marky_0784 | Marky_0783 |  |  |  | Marky_2112 |
| Marinobacter_aquaeolei_VT8_uid59419 |  |  |  |  |  | Maqu_0537 |
| Meiothermus_ruber_DSM_1279_uid46661 | Mrub_1713 | Mrub_1714 |  |  |  | Mrub_2215 |
| Meiothermus_silvanus_DSM_9946_uid49485 | Mesil_2006 | Mesil_2007 |  |  |  | Mesil_0593, Mesil_3586 |
| Methylibium_petroleiphilum_PM1_uid58085 |  |  |  |  |  | Mpe_A3760 |
| Methylobacillus_flagellatus_KT_uid58049 |  |  |  |  |  | Mfla_2107 |
| Methylococcus_capsulatus_Bath_uid57607 |  |  |  |  |  | MCA1537 |
| Methylomicrobium_alcaliphilum_uid77119 | MEALZ_0921 |  |  |  |  | MEALZ_0125 |
| Methylomonas_methanica_MC09_uid67363 | Metme_1144 |  |  |  |  | Metme_4273 |
| Methylotenera_301_uid49469 |  |  |  |  |  | M301_0646 |
| Methylotenera_mobilis_JLW8_uid59373 |  |  |  |  |  | Mmol_0632 |
| Methylovorus_glucosetrophus_SIP3_4_uid59367 |  |  |  |  |  | Msip34_0623 |
| Methylovorus_MP688_uid60723 |  |  |  |  |  | MPQ_0649 |
| Micavibrio_aeruginosavorus_ARL_13_uid73585 |  |  |  |  |  | MICA_1536 |
| Microcystis_aeruginosa_NIES_843_uid59101 |  |  |  |  |  | MAE_04100 |
| Moorella_thermoacetica_ATCC_39073_uid58051 |  |  |  |  |  | Moth_1553 |
| Moraxella_catarrhalis_RH4_uid48809 |  |  |  |  |  | MCR_0335 |
| Myxococcus_fulvus_HW_1_uid68443 | LILAB_12945, LILAB_17450, LILAB_21170 | LILAB_17455 | LILAB_30440 | LILAB_28855 | LILAB_02155, LILAB_17425, LILAB_24640, LILAB_32305 | LILAB_12930, LILAB_12935, LILAB_36425 |
| Myxococcus_xanthus_DK_1622_uid58003 | MXAN_1925, MXAN_2694, MXAN_6703 | MXAN_1926 | MXAN_4461 | MXAN_4140 | MXAN_1331, MXAN_1920, MXAN_3376, MXAN_4868 | MXAN_5787, MXAN_6705, MXAN_6706 |
| Natranaerobius_thermophilus_JW_NM_WN_LF_uid59001 |  |  |  |  |  | Nther_2102 |
| Nautilia_profundicola_AmH_uid59345 |  |  |  |  |  | NAMH_1143 |
| Neisseria_gonorrhoeae_FA_1090_uid57611 |  |  |  |  |  | NGO1908 |
| Neisseria_gonorrhoeae_NCCP11945_uid59191 |  |  |  |  |  | NGK_2349 |
| Neisseria_lactamica_020_06_uid60851 |  |  |  |  |  | NLA_0220 |
| Neisseria_meningitidis_053442_uid58587 |  |  |  |  |  | NMCC_0052 |
| Neisseria_meningitidis_alpha14_uid61649 |  |  |  |  |  | NMO_1957 |
| Neisseria_meningitidis_FAM18_uid57825 |  |  |  |  |  | NMC0036 |
| Neisseria_meningitidis_MC58_uid57817 |  |  |  |  |  | NMB0052 |
| Neisseria_meningitidis_Z2491_uid57819 |  |  |  |  |  | NMA0218 |
| Nitratifractor_salsuginis_DSM_16511_uid62183 |  |  |  |  |  | Nitsa_1652 |
| Nitratiruptor_SB155_2_uid58861 |  |  |  |  |  | NIS_1175 |
| Nitrosococcus_halophilus_Nc4_uid46803 |  |  |  |  |  | Nhal_3792 |
| Nitrosococcus_oceani_ATCC_19707_uid58403 |  |  |  |  |  | Noc_3004 |
| Nitrosococcus_watsonii_C_113_uid50331 |  |  |  |  |  | Nwat_3060 |
| Nitrosomonas_AL212_uid55727 |  |  |  |  |  | NAL212_2928 |
| Nitrosomonas_europaea_ATCC_19718_uid57647 |  |  |  |  |  | NE0965 |
| Nitrosomonas_eutropha_C91_uid58363 |  |  |  |  |  | Neut_2338 |
| Nitrosomonas_Is79A3_uid68745 |  |  |  |  |  | Nit79A3_0119 |
| Nocardioides_JS614_uid58149 |  |  |  |  |  | Noca_1126 |
| Nostoc_azollae_0708_uid49725 |  |  |  |  |  | Aazo_1969 |
| Nostoc_PCC_7120_uid57803 |  |  |  |  |  | all2443 |
| Nostoc_punctiforme_PCC_73102_uid57767 |  |  |  |  |  | Npun_R0117 |
| Oceanimonas_GK1_uid81627 |  |  |  |  |  | GU3_15475 |
| Oceanithermus_profundus_DSM_14977_uid60855 | Ocepr_1644 | Ocepr_1645 |  |  |  | Ocepr_0127, Ocepr_2318 |
| Opitutus_terrae_PB90_1_uid58965 |  |  |  |  |  | Oter_3084 |
| Oscillibacter_valericigenes_uid73895 |  |  |  |  |  | OBV_10190 |
| Paenibacillus_JDR_2_uid59021 |  |  |  |  |  | Pjdr2_2099 |
| Paenibacillus_mucilaginosus_3016_uid89377 |  |  |  |  |  | PM3016_6785 |
| Paenibacillus_mucilaginosus_KNP414_uid68311 |  |  |  |  |  | KNP414_07214 |
| Pelobacter_carbinolicus_DSM_2380_uid58241 | Pcar_0379 | Pcar_0378 | Pcar_2316 |  |  | Pcar_0505, Pcar_0871, Pcar_2148 |
| Pelobacter_propionicus_DSM_2379_uid58255 | Ppro_0659, Ppro_2940 | Ppro_2941 | Ppro_0713 |  |  | Ppro_0536,  Ppro_2513 |
| Pelotomaculum_thermopropionicum_SI_uid58877 |  |  |  |  |  | PTH_1135,  PTH_1146 |
| Persephonella_marina_EX_H1_uid58119 |  |  |  |  |  | PERMA_1505 |
| Photobacterium_profundum_SS9_uid62923 |  |  |  |  |  | PBPRA3142 |
| Pirellula_staleyi_DSM_6068_uid43209 |  |  |  |  |  | Psta_0533 |
| Planctomyces_brasiliensis_DSM_5305_uid60583 |  |  |  |  |  | Plabr_2546 |
| Planctomyces_limnophilus_DSM_3776_uid48643 |  |  |  |  |  | Plim_0409 |
| Polaromonas_JS666_uid58207 |  |  |  |  |  | Bpro_0396 |
| Polaromonas_naphthalenivorans_CJ2_uid58273 |  |  |  |  |  | Pnap_0278 |
| Prochlorococcus_marinus_MIT_9211_uid58309 |  |  |  |  |  | P9211_08231 |
| Prochlorococcus_marinus_MIT_9301_uid58437 |  |  |  |  |  | P9301_06901 |
| Prochlorococcus_marinus_MIT_9303_uid58305 |  |  |  |  |  | P9303_00181 |
| Prochlorococcus_marinus_MIT_9312_uid58357 |  |  |  |  |  | PMT9312_1188 |
| Prochlorococcus_marinus_MIT_9313_uid57773 |  |  |  |  |  | PMT0019 |
| Pseudoalteromonas_atlantica_T6c_uid58283 |  |  |  |  |  | Patl_3717 |
| Pseudoalteromonas_haloplanktis_TAC125_uid58431 |  |  |  |  |  | PSHAa2603 |
| Pseudoalteromonas_SM9913_uid61247 |  |  |  |  |  | PSM_A2581 |
| Pseudogulbenkiania_NH8B_uid73423 |  |  |  |  |  | NH8B_4005 |
| Pseudomonas_aeruginosa_LESB58_uid59275 |  |  |  |  |  | PLES_03931 |
| Pseudomonas_aeruginosa_PA7_uid58627 |  |  |  |  |  | PSPA7_0495 |
| Pseudomonas_aeruginosa_PAO1_uid57945 |  |  |  |  |  | PA0395 |
| Pseudomonas_aeruginosa_UCBPP_PA14_uid57977 |  |  |  |  |  | PA14_05180 |
| Pseudomonas_brassicacearum_NFM421_uid66303 |  |  |  |  |  | PSEBR_a5320 |
| Pseudomonas_fluorescens_F113_uid87037 |  |  |  |  |  | PSF113_5539 |
| Pseudomonas_fluorescens_Pf_5_uid57937 |  |  |  |  |  | PFL_5837 |
| Pseudomonas_fluorescens_Pf0_1_uid57591 |  |  |  |  |  | Pfl01_5318 |
| Pseudomonas_fluorescens_SBW25_uid62971 |  |  |  |  |  | PFLU5762 |
| Pseudomonas_fulva_12_X_uid67351 |  |  |  |  |  | Psefu_0308 |
| Pseudomonas_mendocina_NK_01_uid66299 |  |  |  |  |  | MDS_4475 |
| Pseudomonas_mendocina_ymp_uid58723 |  |  |  |  |  | Pmen_4149 |
| Pseudomonas_stutzeri_A1501_uid58641 |  |  |  |  |  | PST_3965 |
| Pseudomonas_stutzeri_ATCC_17588___LMG_11199_uid68749 |  |  |  |  |  | PSTAB_3924 |
| Pseudomonas_syringae_B728a_uid57931 |  |  |  |  |  | Psyr_0478 |
| Pseudomonas_syringae_phaseolicola_1448A_uid58099 |  |  |  |  |  | PSPPH_0469 |
| Pseudomonas_syringae_tomato_DC3000_uid57967 |  |  |  |  |  | PSPTO_5045 |
| Pseudoxanthomonas_spadix_BD_a59_uid75113 |  |  |  |  |  | DSC_13250 |
| Pseudoxanthomonas_suwonensis_11_1_uid62105 |  |  |  |  |  | Psesu_2063 |
| Psychrobacter_arcticus_273_4_uid58021 |  |  |  |  |  | Psyc_0267 |
| Psychrobacter_cryohalolentis_K5_uid58373 |  |  |  |  |  | Pcryo_0293 |
| Psychrobacter_PRwf_1_uid58459 |  |  |  |  |  | PsycPRwf_1884 |
| Psychromonas_ingrahamii_37_uid58521 |  |  |  |  |  | Ping_3039 |
| Ralstonia_eutropha_H16_uid62925 |  |  |  |  |  | H16_A3104 |
| Ralstonia_eutropha_JMP134_uid58047 |  |  |  |  |  | Reut_A2800 |
| Ralstonia_pickettii_12D_uid58859 |  |  |  |  |  | Rpic12D_2506 |
| Ralstonia_pickettii_12J_uid58737 |  |  |  |  |  | Rpic_2918 |
| Ralstonia_solanacearum_CFBP2957_uid50545 |  |  |  |  |  | RCFBP_10765 |
| Ralstonia_solanacearum_GMI1000_uid57593 |  |  |  |  |  | RSc2682 |
| Ralstonia_solanacearum_PSI07_uid50539 |  |  |  |  |  | RPSI07_0830 |
| Ramlibacter_tataouinensis_TTB310_uid68279 |  |  |  |  |  | Rta_04730 |
| Rhodoferax_ferrireducens_T118_uid58353 |  |  |  |  |  | Rfer_3878 |
| Rhodopirellula_baltica_SH_1_uid61589 |  |  |  |  |  | RB12773 |
| Roseiflexus_castenholzii_DSM_13941_uid58287 | Rcas_4339 | Rcas_1272, Rcas_4340 |  |  |  |  |
| Roseiflexus_RS_1_uid58523 | RoseRS_0200 | RoseRS_0199, RoseRS_0519 |  |  |  |  |
| Rubrobacter_xylanophilus_DSM_9941_uid58057 |  |  |  |  |  | Rxyl_1165 |
| Ruminococcus_albus_7_uid51721 |  |  |  |  |  | Rumal_2390 |
| Saccharophagus_degradans_2_40_uid57921 |  |  |  |  |  | Sde_3640 |
| Sanguibacter_keddieii_DSM_10542_uid40845 |  |  |  |  |  | Sked_17750 |
| Shewanella_amazonensis_SB2B_uid58257 |  |  |  |  |  | Sama_2476 |
| Shewanella_ANA_3_uid58347 |  |  |  |  |  | Shewana3_1195 |
| Shewanella_baltica_OS155_uid58259 |  |  |  |  |  | Sbal_3024 |
| Shewanella_baltica_OS185_uid58743 |  |  |  |  |  | Shew185_3039 |
| Shewanella_baltica_OS195_uid58261 |  |  |  |  |  | Sbal195_3182 |
| Shewanella_baltica_OS223_uid58775 |  |  |  |  |  | Sbal223_1339 |
| Shewanella_baltica_OS678_uid50553 |  |  |  |  |  | Sbal678_3188 |
| Shewanella_denitrificans_OS217_uid58263 |  |  |  |  |  | Sden_2681 |
| Shewanella_frigidimarina_NCIMB_400_uid58265 |  |  |  |  |  | Sfri_2859 |
| Shewanella_halifaxensis_HAW_EB4_uid59007 |  |  |  |  |  | Shal_1173 |
| Shewanella_loihica_PV_4_uid58349 |  |  |  |  |  | Shew_1138 |
| Shewanella_MR_4_uid58345 |  |  |  |  |  | Shewmr4_1194 |
| Shewanella_MR_7_uid58343 |  |  |  |  |  | Shewmr7_1265 |
| Shewanella_oneidensis_MR_1_uid57949 |  |  |  |  |  | SO_3351 |
| Shewanella_pealeana_ATCC_700345_uid58705 |  |  |  |  |  | Spea_1128 |
| Shewanella_piezotolerans_WP3_uid58745 |  |  |  |  |  | swp_3672 |
| Shewanella_putrefaciens_CN_32_uid58267 |  |  |  |  |  | Sputcn32_2686 |
| Shewanella_sediminis_HAW_EB3_uid58835 |  |  |  |  |  | Ssed_1233 |
| Shewanella_violacea_DSS12_uid47085 |  |  |  |  |  | SVI_1044 |
| Shewanella_W3_18_1_uid58341 |  |  |  |  |  | Sputw3181_1325 |
| Shewanella_woodyi_ATCC_51908_uid58721 |  |  |  |  |  | Swoo_1333 |
| Sideroxydans_lithotrophicus_ES_1_uid46801 |  |  |  |  |  | Slit_2759 |
| Sorangium_cellulosum__So_ce_56__uid61629 | sce0386, sce1492, sce2413, sce7249 | sce7248 | sce0012 | sce0777 | sce2917 | sce4468,  sce5323,  sce5324 |
| Sphaerobacter_thermophilus_DSM_20745_uid41997 | Sthe_1568 | Sthe_1567 |  |  |  |  |
| Stenotrophomonas_maltophilia_JV3_uid72473 |  |  |  |  |  | BurJV3_0926 |
| Stenotrophomonas_maltophilia_K279a_uid61647 |  |  |  |  |  | Smlt1089 |
| Stenotrophomonas_maltophilia_R551_3_uid58657 |  |  |  |  |  | Smal_0931 |
| Stigmatella_aurantiaca_DW4_3_1_uid52561 | STAUR_1215, STAUR_2690, STAUR_3438 | STAUR_2691 | STAUR_4816 | STAUR_4587 | STAUR_1958, STAUR_2685, STAUR_3842, STAUR_5654 | STAUR_1211, STAUR_1212, STAUR_6457, STAUR_8051 |
| Streptococcus_sanguinis_SK36_uid58381 |  |  |  |  |  | SSA_2317 |
| Sulfuricurvum_kujiense_DSM_16994_uid60789 |  |  |  |  |  | Sulku_1170 |
| Sulfurihydrogenibium_azorense_Az_Fu1_uid58121 | SULAZ_1272 | SULAZ_1271 |  |  |  | SULAZ_0505 |
| Sulfurihydrogenibium_YO3AOP1_uid58855 |  |  |  |  |  | SYO3AOP1_1744 |
| Sulfurimonas_autotrophica_DSM_16294_uid53043 |  |  |  |  |  | Saut_1367 |
| Sulfurimonas_denitrificans_DSM_1251_uid58185 |  |  |  |  |  | Suden_1251 |
| Sulfurospirillum_deleyianum_DSM_6946_uid41861 |  |  |  |  |  | Sdel_0580 |
| Sulfurovum_NBC37_1_uid58863 |  |  |  |  |  | SUN_0485 |
| Synechococcus_CC9311_uid58123 |  |  |  |  |  | sync_0020 |
| Synechococcus_CC9605_uid58319 |  |  |  |  |  | Syncc9605_0019 |
| Synechococcus_elongatus_PCC_6301_uid58235 |  |  |  |  |  | syc2023_d |
| Synechococcus_elongatus_PCC_7942_uid58045 |  |  |  |  |  | Synpcc7942_2070 |
| Synechococcus_JA_2_3B_a_2_13__uid58537 |  |  |  |  |  | CYB_2142 |
| Synechococcus_JA_3_3Ab_uid58535 |  |  |  |  |  | CYA_1966 |
| Synechococcus_PCC_7002_uid59137 |  |  |  |  |  | SYNPCC7002_A0697 |
| Synechococcus_RCC307_uid61609 |  |  |  |  |  | SynRCC307_0019 |
| Synechococcus_WH_7803_uid61607 |  |  |  |  |  | SynWH7803_0020 |
| Synechocystis_PCC_6803_uid57659 |  |  |  |  |  | slr0161 |
| Syntrophobacter_fumaroxidans_MPOB_uid58177 | Sfum_0318, Sfum_1654 | Sfum_0317, Sfum_1653 |  |  |  | Sfum_1695 |
| Syntrophomonas_wolfei_Goettingen_uid58179 |  |  |  |  |  | Swol_0532 |
| Syntrophothermus_lipocalidus_DSM_12680_uid49527 |  |  |  |  |  | Slip_1188 |
| Syntrophus_aciditrophicus_SB_uid58539 |  |  |  |  |  | SYN_00125 |
| Teredinibacter_turnerae_T7901_uid59267 |  |  |  |  |  | TERTU_0225 |
| Terriglobus_saanensis_SP1PR4_uid53251 |  |  |  |  |  | AciPR4_4210 |
| Thauera_MZ1T_uid58987 |  |  |  |  |  | Tmz1t_3703 |
| Thermaerobacter_marianensis_DSM_12885_uid61727 |  |  |  |  |  | Tmar_0662 |
| Thermanaerovibrio_acidaminovorans_DSM_6589_uid41925 |  |  |  |  |  | Taci_1054 |
| Thermincola_potens_JR_uid48823 |  |  |  |  |  | TherJR_1753 |
| Thermoanaerobacter_brockii_finnii_Ako_1_uid55639 |  |  |  |  |  | Thebr_1095 |
| Thermoanaerobacter_italicus_Ab9_uid46241 |  |  |  |  |  | Thit_1057 |
| Thermoanaerobacter_mathranii_A3_uid49481 |  |  |  |  |  | Tmath_1112 |
| Thermoanaerobacter_pseudethanolicus_ATCC_33223_uid58339 |  |  |  |  |  | Teth39_1068 |
| Thermoanaerobacter_tengcongensis_MB4_uid57813 |  |  |  |  |  | TTE1263 |
| Thermoanaerobacter_wiegelii_Rt8_B1_uid52581 |  |  |  |  |  | Thewi_1212 |
| Thermoanaerobacter_X513_uid53065 |  |  |  |  |  | Thet_1398 |
| Thermoanaerobacter_X514_uid58589 |  |  |  |  |  | Teth514_1505 |
| Thermoanaerobacterium_thermosaccharolyticum_DSM_571_uid51639 |  |  |  |  |  | Tthe_1278 |
| Thermoanaerobacterium_xylanolyticum_LX_11_uid63163 |  |  |  |  |  | Thexy_1156 |
| Thermocrinis_albus_DSM_14484_uid46231 | Thal_1033, Thal_1140 | Thal_1141 |  |  |  | Thal_1039 |
| Thermodesulfatator_indicus_DSM_15286_uid68285 | Thein_0408 | Thein_0407 |  |  |  | Thein_2143 |
| Thermodesulfobacterium_OPB45_uid68283 |  |  |  |  |  | TOPB45_1513 |
| Thermodesulfobium_narugense_DSM_14796_uid66601 |  |  |  |  |  | Thena_0305 |
| Thermodesulfovibrio_yellowstonii_DSM_11347_uid59257 |  |  |  |  |  | THEYE_A2087 |
| Thermomicrobium_roseum_DSM_5159_uid59341 | trd_0864 |  |  |  |  |  |
| Thermosediminibacter_oceani_DSM_16646_uid51421 |  |  |  |  |  | Toce_1929 |
| Thermosipho_africanus_TCF52B_uid59095 |  |  |  |  |  | THA_1712 |
| Thermosipho_melanesiensis_BI429_uid58683 |  |  |  |  |  | Tmel_1396 |
| Thermosynechococcus_elongatus_BP_1_uid57907 |  |  |  |  |  | tll0121 |
| Thermotoga_lettingae_TMO_uid58419 |  |  |  |  |  | Tlet_0381 |
| Thermotoga_maritima_MSB8_uid57723 |  |  |  |  |  | TM1362 |
| Thermotoga_naphthophila_RKU_10_uid42777 |  |  |  |  |  | Tnap_1441 |
| Thermotoga_neapolitana_DSM_4359_uid59065 |  |  |  |  |  | CTN_1229 |
| Thermotoga_petrophila_RKU_1_uid58655 |  |  |  |  |  | Tpet_1421 |
| Thermotoga_RQ2_uid58935 |  |  |  |  |  | TRQ2_1467 |
| Thermotoga_thermarum_DSM_5069_uid68449 |  |  |  |  |  | Theth_1670 |
| Thermovibrio_ammonificans_HB_1_uid62095 | Theam_0113, Theam_1390 | Theam_1391 | Theam_0615 |  |  | Theam_0990 |
| Thermovirga_lienii_DSM_17291_uid77129 |  |  |  |  |  | Tlie_0053,  Tlie_0855 |
| Thermus_scotoductus_SA_01_uid62273 | TSC_c09700 | TSC_c09710 |  |  |  | TSC_c01540 |
| Thermus_thermophilus_HB27_uid58033 | TTC0767 | TTC0766 |  |  |  | TTC1621 |
| Thermus_thermophilus_HB8_uid58223 | TTHA1132 | TTHA1131 |  |  |  | TTHA0365 |
| Thioalkalivibrio_K90mix_uid46181 |  |  |  |  |  | TK90_2414 |
| Thioalkalivibrio_sulfidophilus_HL_EbGr7_uid59179 |  |  |  |  |  | Tgr7_0459,  Tgr7_0460,  Tgr7_2918 |
| Thiobacillus_denitrificans_ATCC_25259_uid58189 |  |  |  |  |  | Tbd_2419 |
| Thiomicrospira_crunogena_XCL_2_uid58183 |  |  |  |  |  | Tcr_1750 |
| Thiomonas_intermedia_K12_uid48825 |  |  |  |  |  | Tint_0337 |
| Tolumonas_auensis_DSM_9187_uid59395 |  |  |  |  |  | Tola_0966 |
| Trichodesmium_erythraeum_IMS101_uid57925 |  |  |  |  |  | Tery_1747 |
| Truepera_radiovictrix_DSM_17093_uid49533 | Trad_1082 |  |  |  |  | Trad_2339 |
| Variovorax_paradoxus_EPS_uid62107 |  |  |  |  |  | Varpa_5474 |
| Variovorax_paradoxus_S110_uid59437 |  |  |  |  |  | Vapar_4813 |
| Verminephrobacter_eiseniae_EF01_2_uid58675 |  |  |  |  |  | Veis_1688 |
| Vibrio_anguillarum_775_uid68057 |  |  |  |  |  | VAA_01656 |
| Vibrio_cholerae_IEC224_uid89389 |  |  |  |  |  | O3Y_02145 |
| Vibrio_cholerae_M66_2_uid59355 |  |  |  |  |  | VCM66_0447 |
| Vibrio_cholerae_MJ_1236_uid59387 |  |  |  |  |  | VCD_001145 |
| Vibrio_cholerae_O1_2010EL_1786_uid78933 |  |  |  |  |  | Vch1786_I2783 |
| Vibrio_cholerae_O1_biovar_El_Tor_N16961_uid57623 |  |  |  |  |  | VC0462 |
| Vibrio_cholerae_O395_uid58425 |  |  |  |  |  | VC0395_A0014 |
| Vibrio_EJY3_uid83161 |  |  |  |  |  | VEJY3_13450 |
| Vibrio_Ex25_uid41601 |  |  |  |  |  | VEA_002458 |
| Vibrio_fischeri_ES114_uid58163 |  |  |  |  |  | VF_0431 |
| Vibrio_fischeri_MJ11_uid58907 |  |  |  |  |  | VFMJ11_0431 |
| Vibrio_furnissii_NCTC_11218_uid82347 |  |  |  |  |  | vfu_A03050 |
| Vibrio_harveyi_ATCC_BAA_1116_uid58957 |  |  |  |  |  | VIBHAR_03577 |
| Vibrio_parahaemolyticus_RIMD_2210633_uid57969 |  |  |  |  |  | VP2615 |
| Vibrio_splendidus_LGP32_uid59353 |  |  |  |  |  | VS_2682 |
| Vibrio_vulnificus_CMCP6_uid62909 |  |  |  |  |  | VV1_1526 |
| Vibrio_vulnificus_MO6_24_O_uid62243 |  |  |  |  |  | VVMO6_00434 |
| Vibrio_vulnificus_YJ016_uid58007 |  |  |  |  |  | VV2874 |
| Wolinella_succinogenes_DSM_1740_uid61591 |  |  |  |  |  | WS0533 |
| Xanthomonas_albilineans_GPE_PC73_uid43163 |  |  |  |  |  | XALc_2160 |
| Xanthomonas_axonopodis_citri_306_uid57889 |  |  |  |  |  | XAC2924 |
| Xanthomonas_axonopodis_citrumelo_F1_uid73179 |  |  |  |  |  | XACM_2855 |
| Xanthomonas_campestris_8004_uid57595 |  |  |  |  |  | XC_1358 |
| Xanthomonas_campestris_ATCC_33913_uid57887 |  |  |  |  |  | XCC2755 |
| Xanthomonas_campestris_B100_uid61643 |  |  |  |  |  | xccb100_1406 |
| Xanthomonas_campestris_vesicatoria_85_10_uid58321 |  |  |  |  |  | XCV3068 |
| Xanthomonas_oryzae_KACC10331_uid58155 |  |  |  |  |  | XOO1418 |
| Xanthomonas_oryzae_MAFF_311018_uid58547 |  |  |  |  |  | XOO_1302 |
| Xanthomonas_oryzae_PXO99A_uid59131 |  |  |  |  |  | PXO_01993 |
| Xylella_fastidiosa_9a5c_uid57849 |  |  |  |  |  | XF1633 |
| Xylella_fastidiosa_M12_uid58763 |  |  |  |  |  | Xfasm12_1253 |
| Xylella_fastidiosa_M23_uid58809 |  |  |  |  |  | XfasM23_1221 |
| Xylella_fastidiosa_Temecula1_uid57869 |  |  |  |  |  | PD1147 |

- Sequence identifiers correspond to locus tags.
