# Supplementary material for: A Response Regulator Interfaces between the Frz Chemosensory System and the MglA/MglB GTPase/GAP Module to Regulate Polarity in Myxococcus xanthus
Source: PLoS Genet. 2012 Sep 13;8(9):e1002951. doi: 10.1371/journal.pgen.1002951 (PMC3441718; doi:10.1371/journal.pgen.1002951)
Supplement: Text S1 — Supplementary Materials and Methods. (DOC) [file pgen.1002951.s011.doc]

**SUPPLEMENTARY MATERIALS AND METHODS**

Protein purification. Overexpression strains expressing His_6_-RomR, His_6_-MglB, MglA-His_6_, MalE, MalE-RomR, GST or GST-MglA were grown in LB containing 100 µg/ml ampicillin. At a cell density of 7×10^8^ cells/ml, protein production was induced by adding 0.1 mM isopropyl-1-thio-ß-D-galactopyranoside (IPTG) for 20h at 18 °C. Cells were harvested by centrifugation at 4.700 rpm, 20 min, 4 °C and resuspended in lysis buffer. For His_6_-RomR, His_6_-MglB, MglA-His_6_ the lysis buffer was: 50 mM NaH_2_PO_4_ pH 8.0, 300 mM NaCl, 10mM imidazole,Protease Inhibitor tablets (Roche), 1mg/ml lysozyme (Merck). For MalE, MalE-RomR, GST, GST-MglA the lysis buffer was: 20 mM Tris/HCl pH 7.5, 300 mM NaCl, 10% glycerol, Protease Inhibitor tablets (Roche), 1mg/ml lysozyme (Merck) Protease Inhibitors, lysozyme. Cells were lysed by ultrasonication and debris removed by centrifugation at 4.700 rpm, 20 min, 4 °C. His_6_-tagged proteins were purified using Ni^++^-NTA columns (Macherey-Nagel), GST-tagged proteins were purified using a glutathione-Sepharose column (Novagen), and MalE-tagged proteins were purified using amylose beads (Biolabs) as recommended by the manufacturers. Elutions were performed with elution buffers containing 50 mM NaH_2_PO_4_ pH 8.0, 300 mM NaCl, 200mM imidazole for His_6_-tagged proteins, 20 mM Tris/HCl pH 7.5, 300 mM NaCl, 10% glycerol, 10 mM glutathione for GST-tagged proteins, and 20 mM Tris/HCl pH 7.5, 300 mM NaCl, 10% glycerol, 10 mM maltose for MalE-tagged proteins. After elution, proteins were dialysed against a storage buffer containing 50 mM NaH_2_PO_4_ pH 8.0, 300 mM NaCl, 10% glycerol for His_6_-tagged proteins, or 20 mM Tris/HCl pH 7.5, 300 mM NaCl, 10% glycerol for GST-tagged or MalE-tagged proteins, and stored at -80 °C. The protein concentration and purity was analyzed using the BioRad Protein assay Kit (Bio-Rad) and SDS-page , respectively.

Plasmid construction:

pSL54: Plasmid for overexpression of MglA-GST*. mglA* was amplified with the primers oMglA-EcoRI and oMglAstop-NotI using chromosomal DNA of *M. xanthus* as a template. The amplified fragment was purified and cloned into pGEX4T-1 with the restriction sites *EcoR*I and *Not*I. To overexpress MglA-GST, the plasmid was transformed into *E. coli* Rosetta2.

pES1: Plasmid for overexpression of His_6_-MglB*. mglB* was amplified with the primers omglB3 and oMglB4 using chromosomal DNA of *M. xanthus* as a template. The amplified fragment was purified and cloned into pet45b+ with the restriction sites *Hind*III and *BamH*I. To overexpress His_6_-MglB, the plasmid was transformed into *E. coli* Rosetta2.

pDK47: Plasmid for overexpression of His_6_-RomR. *romR* was amplified with the primers HisRomRPstI and HisRomRrv using chromosomal DNA of *M. xanthus* as a template. The amplified fragment was purified and cloned into pet45b+ with the restriction sites *Pst*I and *Hind*III. To overexpress His_6_-RomR, the plasmid was transformed into *E. coli* Rosetta2.

pDK28: Plasmid for overexpression of MalE-RomR*. romR* was amplified with the primers MalE-RomRfwand MalE-RomRrv using chromosomal DNA of *M. xanthus* as a template. The amplified fragment was purified and cloned into pMal-c2 with the restriction sites *EcoR*I and *HindI*II. To overexpress MalE-RomR, the plasmid was transformed into *E. coli* Rosetta2.

pSL37: Plasmid for generation of Δ*romR*. The deletion cassette for *romR* was amplified by creating an AB fragment upstream of *romR*, using the primer oDromR-1 and oDromR-2, and a downstream CD fragment using oDromR-3 and oDromR-4 with chromosomal DNA of *M. xanthus* as a template. The two fragments were fused to an AD fragment by overlap PCR, using an overlap in the primers oDromR-2 and oDromR-3. The AD fragment was then cloned into pBJ114 using the restriction sites *EcoR*I and *Hind*III.

pTS08: Plasmid to introduce the point mutation leading to the Q82A substitution in *mglA*. Primers oMglAQ82Aforw and oMglAQ82Arev including the point mutation were used to perform the amplification with the “QuikChange XL Site-Directed Mutagenesis Kit“ (Stratagene, Amsterdam). The plasmid containing *mglA*^Q82A^ was digested with *Hind*III and *EcoR*I and cloned into pBJ114, to introduce the point mutation at the endogenous site by double homologous recombination.

pFD1: Plasmid for generation of Δ*frzZ*. The deletion cassette for *frzZ* was amplified by amplifying an AB fragment upstream of *frzZ*, using the primer FrzZA and FrzZB, and a downstream CD fragment using FrzZC and FrzZD with chromosomal DNA of *M. xanthus* as a template. The two fragments were fused to an AD fragment by overlap PCR, using an overlap in the Primers oDromR-2 and oDromR-3. The AD fragment was then cloned into pBJ114 using the restriction sites *EcoR*I and *Hind*III.

pDK78: Plasmid to generate *mglB-mcherry* fusion expressed from the native site. To construct the plasmid pDK78, three PCR fragments were amplified, the AB fragment, containing the upstream region of *mglB* and *mglB* (MglBfwsur/ MglBrvmcherry), the CD fragment, containing *mcherry* (Mcherryfw/Mcherryrv) and the EF fragment containing the downstream region of *mglB* (MglAfw/ MglAsurrv) using chromosomal DNA of *M. xanthus* as a template and a plasmid containing *mcherry,* respectively. The primer MglBrvmcherry contains a homologous region to Mcherryfw and the primer Mcherryrv contains a homologous region to MglAfw. Therefore overlap PCRs could be performed to create a fragment AF. This fragment was cloned into pBJ114 using the restriction sites *Hind*III and *EcoR*I.

pDK79: The plasmid pDK79 was constructed analogous to pDK78, using chromosomal DNA of *ΔmglA* instead of WT DNA as a template for the EF fragment.

pDK3: Plasmid for generation of *PpilA-romR*^369-420^*-GFP* fusion expressed from the *attB* site. Primers DA3 and DA4 were used, to amplify the fragment for *romR*^369-420^*.* A second PCR was performed using oCrGFP-3 and oCrGFP-2 to amplify *gfp* from a plasmid containing *gfp*. First the two fragments were cloned into pBluescript II SK- using *Xba*I and *EcoR*V for the *romR* fragment, and *EcoR*V and *Hind*III for gfp, creating a C-terminal *gfp* fusion of the fragment. This fusion fragment was then cloned into pSW105 using *Xba*I and *Hind*III.

pDK4: Plasmid for generation of *PpilA-romR*^116-368^*-GFP* fusion expressed from the *attB* site. Primers DA1 and DA2 were used, to amplify the fragment of *romR*^116-368^*.* A second PCR was performed using oCrGFP-3 and oCrGFP-2 to amplify *gfp* from a plasmid containing *gfp*. First the two fragments were cloned into pBluescript II SK- using *Xba*I and *EcoR*V for the *romR* fragment, and *EcoR*V and *Hind*III for *gfp*, creating a C-terminal *gfp* fusion of the fragment. This fusion fragment was then cloned into pSW105 using *Xba*I and *Hind*III.

pDK5 Plasmid for generation of *PpilA-romR*^332-420^*-GFP* fusion expressed from the *attB* site. Primers DA5 and DA4 were used, to amplify the fragment of *romR*^332-420^*.* A second PCR was performed using oCrGFP-3 and oCrGFP-2 to amplify *gfp* from a plasmid containing *gfp*. First the two fragments were cloned into pBluescript II SK- using *Xba*I and *EcoR*V for the *romR* fragment, and *EcoR*V and *Hind*III for *gfp*, creating a C-terminal *gfp* fusion of the fragment. This fusion fragment was then cloned into pSW105 using *Xba*I and *Hind*III.

pDK6 Plasmid for generation of *PpilA-romR*^116-420^*-GFP* fusion expressed from the *attB* site. Primers DA1 and DA4 were used, to amplify the fragment of *romR*^116-420^*.* A second PCR was performed using oCrGFP-3 and oCrGFP-2 to amplify *gfp* from a plasmid containing *gfp*. First the two fragments were cloned into pBluescript II SK- using *Xba*I and *EcoR*V for the *romR* fragment, and *EcoR*V and *Hind*III for *gfp*, creating a C-terminal *gfp* fusion of the fragment. This fusion fragment was then cloned into pSW105 using *Xba*I and *Hind*III.

**References**

1. Sambrook J, Russell DW (2001) Molecular cloning : a laboratory manual. Cold Spring Harbor, N.Y.: Cold Spring Harbor Laboratory Press.
